# Identification of *RECK* as an evolutionarily conserved tumor suppressor gene for zebrafish malignant peripheral nerve sheath tumors

**DOI:** 10.18632/oncotarget.25236

**Published:** 2018-05-04

**Authors:** Rashmi Kumari, Martin R. Silic, Yava L. Jones-Hall, Alexandra Nin-Velez, Jer-Yen Yang, Suresh K. Mittal, GuangJun Zhang

**Affiliations:** ^1^ Department of Comparative Pathobiology, Purdue University, West Lafayette, Indiana 47907, USA; ^2^ Purdue Institute for Inflammation, Immunology and Infectious Disease (PI4D), Purdue University, West Lafayette, Indiana 47907, USA; ^3^ Department of Basic Medical Sciences, Purdue University, West Lafayette, Indiana 47907, USA; ^4^ Purdue University Center for Cancer Research, Purdue University, West Lafayette, Indiana 47907, USA; ^5^ Purude Institute for Integrative Neuroscience, Purdue University, West Lafayette, Indiana 47907, USA

**Keywords:** RECK, tumor suppressor gene (TSG), MPNST, pPNET, zebrafish

## Abstract

Malignant peripheral nerve sheath tumors (MPNSTs) are a type of sarcoma with poor prognosis due to their complex genetic changes, invasive growth, and insensitivity to chemo- and radiotherapies. One of the most frequently lost chromosome arms in human MPNSTs is chromosome 9p. However, the cancer driver genes located on it remain largely unknown, except the tumor suppressor gene, p16 *(INK4)/CDKN2A*. Previously, we identified *RECK* as a tumor suppressor gene candidate on chromosome 9p using zebrafish-human comparative oncogenomics. In this study, we investigated the tumorigenesis of the *reck* gene using zebrafish genetic models in both *tp53* and *ribosomal protein* gene mutation background. We also examined the biological effects of *RECK* gene restoration in human MPNST cell lines. These results provide the first genetic evidence that *reck* is a *bona fide* tumor suppressor gene for MPNSTs in zebrafish. In addition, restoration of the *RECK* gene in human MPNST cells leads to growth inhibition suggesting that the reactivation of RECK could serve as a potential therapeutic strategy for MPNSTs.

## INTRODUCTION

Malignant peripheral nerve sheath tumors (MPNSTs) are a type of sarcoma that originate from the neural crest/Schwann cell lineage in humans, and have a poor prognosis due to their complex genetic changes, invasive growth nature, and insensitiveness to chemo- and radiotherapies [1–3]. For the etiology of MPNST, only a few cancer genes are currently known to be related to this type of malignancy: *NF1*, *NF2*, *SMARCB1* and *LZTR1* [4, 5]. Surgical resection is the mainstay of treatment, but often compromised by many factors such as distant metastasis, local damage, and inoperable locations [6]. Moreover, there is currently no targeted therapy available for this type of malignancy, mainly due to the lack of knowledge of its cancer driver genes.

Cancer driver genes are a small number of genes found mutated in cancer genomes, and their mutations biologically contribute to cancer initiation and progression [7]. The remaining mutated genes in the cancer genome are defined as passenger genes, if their mutations have no impact on tumorigenesis. Based on gain or loss of function, cancer driver genes can be divided into either oncogenes or tumor suppressor genes (TSGs). One of the goals of current cancer research is to identify all the cancer driver genes, so that they can be pursued as new targets for the development of cancer therapy. Targeted cancer therapy has emerged as a better alternative to other treatment options because of its high specificity and fewer side effects. Effective targeted cancer therapies require accurate identification of the targetable cancer driver genes.

Identification of cancer driver genes is difficult, due to the plethora of mutations in the cancer cell genomes and the time required for functional validation. One of the extreme situations is represented with aneuploid chromosomes, which are found in more than 90% of solid tumors and 75% of hematopoietic tumors [8]. This difficulty stems from the large number of genes on the chromosome arm. Even if the driver genes have no mutations, it is not possible to distinguish driver from passenger genes despite advances in modern technologies, such as parallel sequencing and microarray. Cross-species comparative oncogenomics emerged as a solution to pinpoint cancer driver candidates, as the cancer driver genes tend to be enriched in common genetic mutations, due to the gene functional conservation. This approach has already been demonstrated to be effective in mice and dogs [9–12]. The research community has recently showed that zebrafish-human comparative oncogenomics is particularly effective with large aneuploid chromosomes because gene synteny was extensively re-organized during vertebrate evolution [13–15]. Furthermore, the zebrafish model provides a valuable system for functional studies of candidate drivers from omics studies. It has been well-established that zebrafish tumors share many similarities with humans including histology, etiology of genetic mutations (*tp53*, *pten*, etc.), the transcriptome, and copy number alterations (CNAs) [16, 17].

Chromosome 9p is one of the most frequently lost chromosome arms in human MPNSTs and many other types of solid tumors [18–24]. *CDKN2A/p16/INK4* is one of the currently known TSGs on this chromosome arm, but this does not exclude the existence of other TSGs on 9p. Using zebrafish-human comparative oncogenomics, on human chromosome 9p, we have identified the *RECK* gene as a cancer driver candidate that could potentially be a tumor suppressor gene for MPNSTs [13]. *RECK* is located on chromosome 9p, which is frequently lost in human MPNSTs [25]. The *RECK* (reversion-inducing cysteine-rich protein with Kazal motifs) gene encodes a 110 kDa membrane-bound glycoprotein [26]. It was discovered as a gene that was able to rescue the aberrant cell morphology of RAS-transformed fibroblasts [27]. The *Reck* gene is expressed in many mouse tissues, and it was found to be down-regulated in many types of human cancers such as the liver, pancreatic, breast, colon, lung, melanoma, and fibrosarcoma [26, 28]. Low expression of *RECK* was reported to correspond to biological malignancy, such as invasiveness and metastasis [26, 29, 30]. Thus, the *RECK* gene was believed to be a tumor suppressor gene, but *in vivo* experimental evidence was missing to support this hypothesis. Knockout mice indicate that *Reck* is a developmentally essential gene and is critical for angiogenesis through regulating matrix metalloproteinases [31]. These mouse embryos die at stage E10.5 in the homozygous knockouts, due to hemorrhage and neuronal defects [31]. Moreover, there is no report of spontaneous tumors in the heterozygous *RECK* knockout mice. Whether the *Reck* gene functions as a TSG *in vivo* remains unknown, although it is worth noting that evidence from cell culture models uphold this hypothesis [26].

Here, we performed functional genetic studies on the zebrafish *reck* gene, and provided the first evidence that zebrafish *reck* is a tumor suppressor gene *in vivo*.

## RESULTS

**Figure 1:**
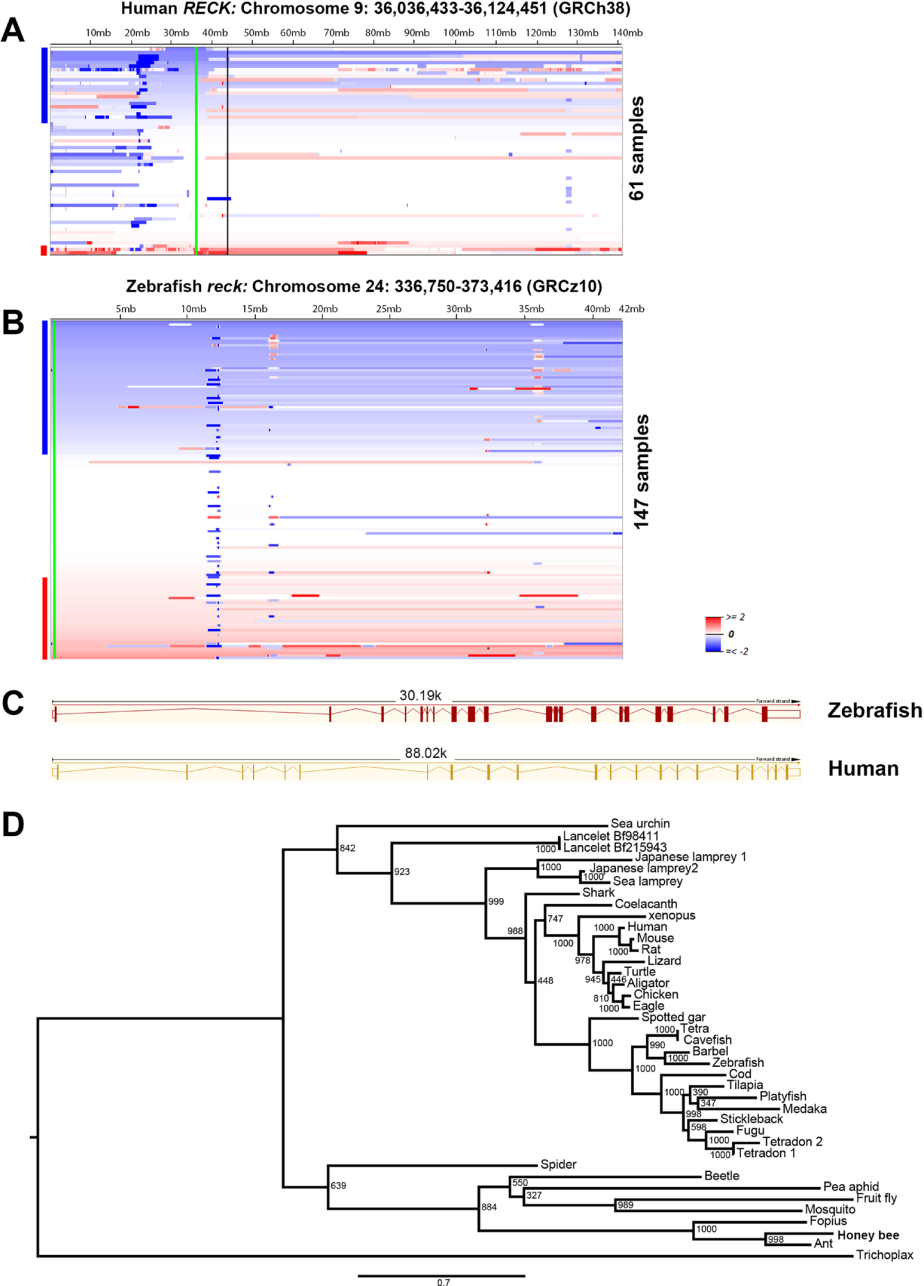
Frequent loss of DNA copy numbers of the *RECK* gene in human and zebrafish MPNSTs (**A**) Heat map of human chromosome 9 is shown. *RECK* is located on chromosome 9p, which is found to be under-represented in 39% (24 out of 61) of human MPNSTs [13]. The green line represents the *RECK* gene locus, and the vertical black line indicates the centromere of chromosome 9. The human *RECK* gene location and reference genome assembly are labeled above the heat map. (**B**) Zebrafish *reck* is located on chromosome 24, which is under-represented in about 39% (58 out of 147) of zebrafish MPNSTs. Samples are sorted top-to-bottom by decreasing deletion amplitude at their respective *RECK/reck* locus, indicated by a green line. Blue and red bars at the right side of each panel indicate samples with *RECK/reck,* losses (blue) or gains (red). Color densities are corresponding to the degree of loss and gain as previously described [13]. The zebrafish *reck* gene location and reference genome assembly are labeled above the heat map. (**C**) Human and zebrafish *RECK* have different intron-exon structures, but both are composed of 21 exons. The solid red/orange vertical boxes represent the gene coding regions, and the empty boxes indicate un-transcribed regions. Red/orange thin lines indicate the introns. Human *RECK* transcript: *RECK*-001 ENST00000377966.3. Zebrafish *reck* transcript: *reck*-001 ENSDART00000129135.2. (**D**) Maximum likelihood phylogeny of RECK proteins, as obtained with JTT plus gamma distances (α = 0.941). Numbers around each node indicate bootstrap values based on 1,000 replicates. Branch lengths are proportional to expected replacements per site. The tree was rooted with placozoa *(Trichoplax adhaerens)*.

Zebrafish are becoming a popular cancer model, due to their tractable genetics and conserved vertebrate biology similar to humans [16]. In zebrafish, MPNSTs can be initiated with *tp53* homozygous point mutations [32], or heterozygous ribosomal protein gene (*rp*) mutations [33]. Previously, we have found that zebrafish and human MPSNTs share high similarities, such as histology, aneuploidy, and copy number alterations [13, 14, 32, 33]. To investigate whether *RECK* is a *bona fide* tumor suppressor gene in MPNSTs, we took advantage of the zebrafish genetic model. First, we re-analyzed our previous DNA copy number data from both zebrafish and human MPNSTs [13]. In both human and zebrafish MPNSTs, about 39% of tumors lose DNA copy numbers of the *RECK* gene (Figure 1A–1B). As the zebrafish genome usually contains duplicates for some of the genes, due to teleost whole genome duplication, we performed a BLAT search using the human *RECK* gene as a bait in both NCBI and Ensembl. Both searches suggested that a single copy of the *reck* gene is present in the zebrafish genome. The zebrafish Reck shares 65% protein sequence similarity with human RECK, both proteins are coded by 21 exons (Figure 1C), and both feature a KAZAL_FS (Kazal type serine protease inhibitor and follistatin-like) domain. To further demonstrate the zebrafish *reck* gene identity, we conducted phylogenetic analysis using protein sequences from representative species. Our maximum likelihood phylogenetic analysis revealed that *RECK* is a conserved gene in metazoans (Figure 1D), indicating that zebrafish *reck* may have similar biological functions with the human *RECK* gene.

The zebrafish *reck* gene is found to be expressed in the developing neural crest cells, and is required for appropriate sensory neuron formation in a cell-autonomous fashion [34]. Since MPNST is a type of malignancy of Schwann cells that originate from the neural crest, we first validated the expression of the zebrafish *reck* gene by *In Situ* hybridization during zebrafish embryogenesis. Indeed, as reported, we confirmed that reck is expressed in the developing neural crest cells (Supplementary Figure 1), suggesting it has a developmental role in the neural crest-derived cells.

A TSG is defined as a gene whose inactivation leads to tumor formation, and promotes tumor development *in vivo*. To functionally evaluate whether *reck* is a *bona fide* TSG in zebrafish MPNSTs, we took advantage of the currently available *reck* loss-of-function mutant, *sensory deprived* (*sdp)* [34]. If a TSG is strong enough, we can expect the development of spontaneous tumors in the loss-of-function mutant animals in a relatively short time. However, if a TSG is weak, we may need a longer follow-up time for tumorigenesis requiring a large number of animals. Based on the principle of genetic synthetic/additive effect, we hypothesized that loss of *rec*k will accelerate tumorigenesis in a *tp53* or *ribosomal protein* mutant background, as both types of mutation were known to cause spontaneous MPNSTs in zebrafish [32, 33].

**Figure 2:**
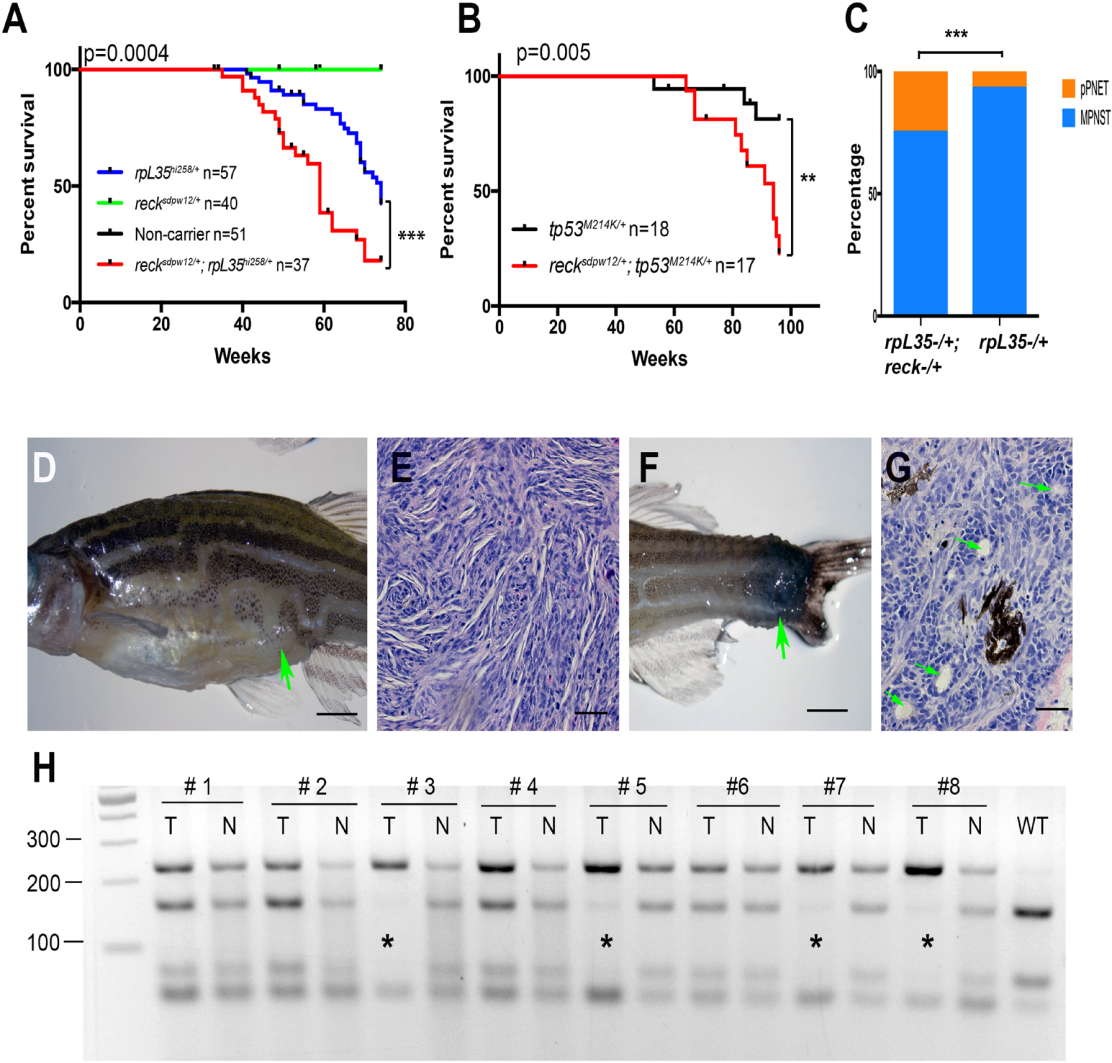
*Reck* is a tumor suppressor gene in zebrafish MPNSTs (**A–B**) Loss of the *reck* gene function facilitates tumorigenesis in either *rp* or *tp53* mutant background. Kaplan–Meier curves showing tumor-free survival of cohorts of single and double heterozygotes derived from *rpL35^hi258/+^* x *sdp^w12/+^* crosses (A) and *tp53^M214K/+^* x *sdp^w12/+^* crosses (B). Fish from all crosses were genotyped by PCR for each relevant mutation at 6–8 weeks of age, and housed segregated by genotype. In all panels, the numbers of fish (n) of each genotype are shown next to the genotype. The p values between the *rp* or *tp53* single heterozygote and the compound mutants are shown in the figures. The green line (*sdp^w12/+^*) and black line (non-carrier) are overlapped in panel (A). (**C**) More zebrafish developed pPNETs in the *rpL35^hi258/+^*;*sdp^w12/+^* compared to *rpL35^hi258/+^*. Two-tailed fisher's exact test was used for statistical analysis. *p* = 0.008. Asterisks indicate statistical significance (A–C). (**D**) A typical zebrafish with abdominal MNPST. The green arrow indicates the position of the tumor. (**E**) Hematoxylin and Eosin (H&E) staining showed typical histopathology of zebrafish MPNST, which is characterized by interlacing bundles and swirls of spindle shaped tumor cells. (**F**) A typical pPNET that was found in the tail region. The green arrow indicates the position of the tumor. (**G**) Representative examples of pPNET histology by H&E staining. The pseudorosettes (Flexner-Wintersteiner rosettes) are indicated with green arrows. (**H**) The wildtype allele is lost in some of the *tp53^M214K/+^*;*sdp^w12/+^* tumors (tumor samples are labeled with asterisks). The *reck* gene was amplified by PCR using primers flanking the *sdp^w12^* mutation site. The PCR product was then digested with Ava II, which only cuts the 233bps wildtype PCR product into 158bps and 75bps fragments [34]. T, tumor; N, normal tissue from caudal fin. Note, the lowest band is from the PCR primer polymerization. Scaler bar = 250 mm (D, F) Scale bar = 50 mm (E, G).

To test this hypothesis, we crossed the *reck* mutant, *spd^w12/+^,* with the *tp53* or *ribosomal protein L35 (rpL35)* mutant, and tracked the development of tumors. In zebrafish, 17 *rp* gene mutants were known to cause MPNSTs and the impact of these genetic mutations was believed to be similar [33, 35, 36]. Here, we chose the *rpL35* mutant, hi258, as a representative of the *rp* TSGs. As expected, the *reck* mutation accelerated tumor formation rate in both mutant backgrounds (Figure 2A–2B), demonstrating that *reck* is indeed a *bona fide* TSG. Interestingly, we noticed that there was a tumor spectrum shift in the *reck* and *rpL35* fish cross (Figure 2C). Although most tumors were MPNSTs (Figure 2D–2E), 25% (6 out of 24) of tumors were peripheral primitive neuroectodermal tumors (pPNETs, Figure 2F–2G) in *sdp^w12/+^;rpL35^hi258/+^*, while only 7% (2 out of 28) were pPNETs in *rpL35^hi258/+^*, suggesting that the *reck* gene may have a cell type differential role during tumor formation. As we did not detect any visible tumors in the *reck* heterozygotes (*sdp^w12/+^*) in either cross, we reasoned that *reck* may function as a TSG in a two-hit paradigm manner, exemplified by the *RB1* gene [37]. To examine this possibility, we investigated the loss of heterozygosity (LOH) in a few tumor-tail paired tissue samples. Indeed, we found 4 out of 8 cases where the wildtype allele was lost in these tumors (Figure 2H). Altogether, our results supported the hypothesis that *reck* is a TSG that may follow the two-hit paradigm of tumor suppression.

**Figure 3:**
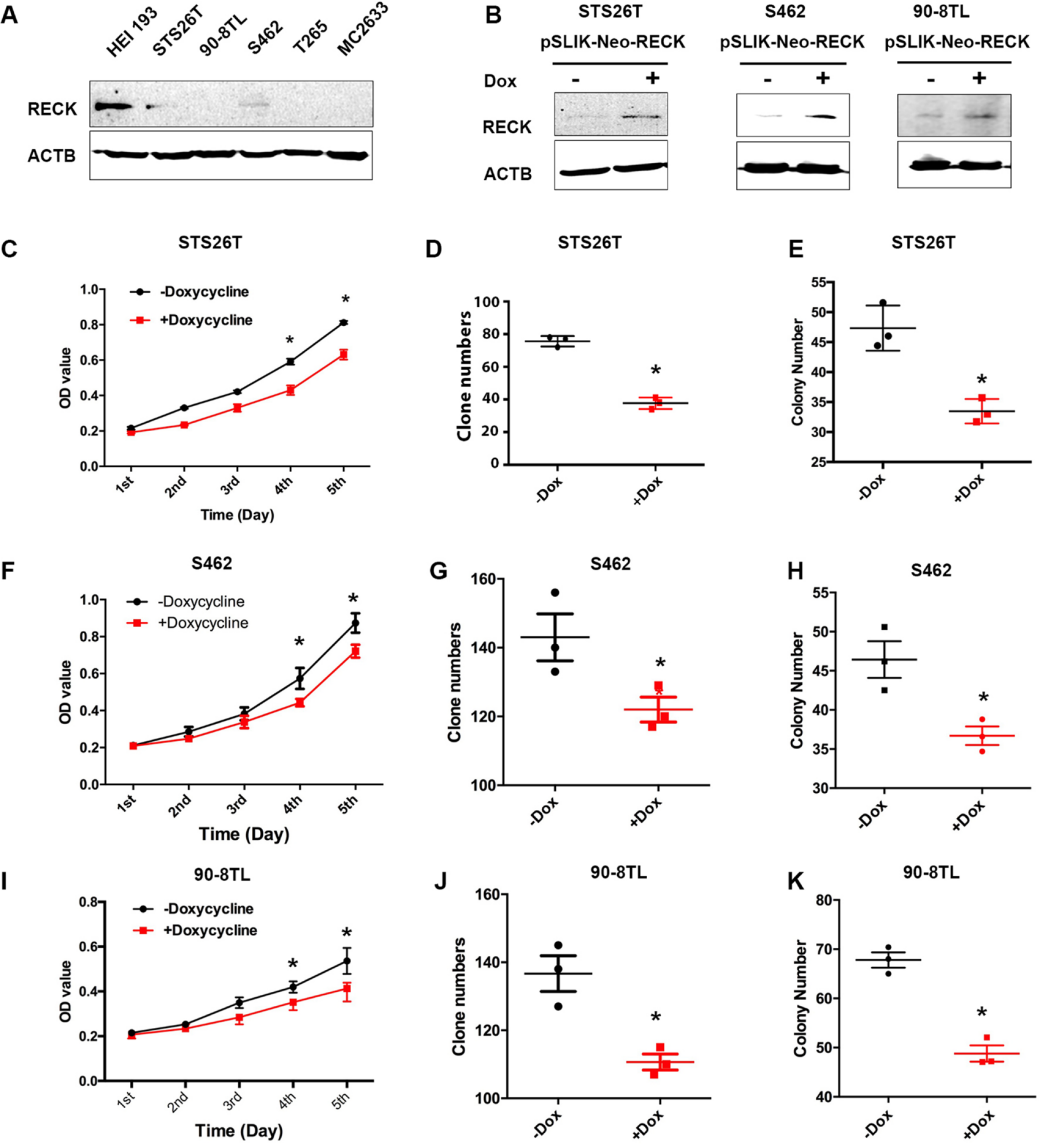
Restoration of *RECK* in human MPNST cells suppresses cell growth (**A**) RECK expression is lost or dramatically decreased in human MPNST cells (STS26T, 90-8TL, S462, T265T and MC2633) compared to a benign neurofibroma cell line, HEI193. (**B**) RECK expression was restored in lentiviral RECK-stable cells (STS26T, S462 and 90-8TL) induced by doxycycline (1 μg/ml) shown by Western blots. (**C**, **F**, **I**) MTT assays revealed that cell growth was inhibited by *RECK* restoration in STS26T, S462 and 90-8TL cell lines, respectively. Growth curves were created using the optical density values collected over five days. (**D**, **G**, **J**) Independent cell growth by plate colony formation assays on STS26T, S462 and 90-8TL, respectively. Crystal violet stained cell colonies (diameter ≥ 0.2 mm) were counted. Asterisk indicated the statistical significance by student *t*-test, *p* < 0.05. (**E**, **H**, **K**) Anchorage- independent growth by soft agar assay. The cells were seeded on agarose plates for colony formation assays, and colonies whose diameters are larger than 20um were counted two weeks later. Asterisks indicated the statistical significance by student *t*-test, *p* < 0.05.

Since the DNA copy number of *RECK* is frequently lost in human MPNSTs, we hypothesized that protein levels of RECK in human MPNST cell lines are generally decreased. To test this, we performed Western blots and found that RECK was not detectable or expressed at a lower level in human MPNST cells, compared to the benign neurofibroma cell line, HEI193 (Figure 3A). TSG restoration was found to inhibit tumor growth and reverse other cancer characteristics [38], therefore, we reasoned that restoration of *RECK* in human MPNST cells would inhibit cell growth. To demonstrate this, we first generated three tetracycline-inducible stable human MPNST cell lines using an untagged full-length *RECK* (pSLIK-neo-*RECK*) construct (Figure 3B). We chose STS26T (*TP53* mutant), S462 (*NF1* mutant), and 90-8TL (*NF1* micro-deletion) human MPNST cell lines, as *TP53* and *NF1* are commonly mutated in human MPNSTs [39, 40]. Moreover, we previously found that doxycycline has no impact on the growth of these cell lines [41]. Next, we examined cell growth by MTT, colony formation assays, and soft agar anchorage-independent growth assays. Indeed, upon restoration of RECK expression the cell growth rates were decreased in all three MPNST cell lines (Figure 3C, 3F, 3I). Similarly, growth inhibition effects were also observed in the colony formation assay (Figure 3D, 3G, 3J) and the soft agar anchorage-independent growth assay (Figure 3E, 3H, 3K), suggesting that *RECK* functions as a TSG in human MPNST cells. Notably, this growth inhibition is not dependent on TP53 or NF1, suggesting that RECK is a mechanistically independent TSG.

## DISCUSSION

The *RECK* gene is one of the TSG candidates on human chromosome 9p in MPNSTs and other types of tumors [18–24]. Although, accumulating evidence from cellular experiments indicates that the *RECK* gene is a TSG *in vivo*, there is no clear genetic evidence to prove this notion, since this gene is a developmentally essential gene in mouse [26, 31]. Here, our results provide the first *in vivo* evidence that *reck* is a *bona fide* tumor suppressor gene in zebrafish MPNSTs. Moreover, we validate that the restoration of RECK leads to inhibition of human MPNST cell growth.

In our genetic experiments, the loss of one *reck* gene allele indeed accelerated tumorigenesis initiated with *tp53*–/–, or *rpL35^hi258/+^*, although we did not find spontaneous tumors in zebrafish *reck* mutant, *sdp^w12/+^*, within 2 years. These genetic synthetic/additive effects between *reck* and *tp53* or *rpL35* suggested that *reck i*s a TSG and it is not in the same genetic pathway of *tp53* or *rpL35*, *i.e.* there is no direct genetic interactions. It is worth to note that the heterozygous *reck^sdpw12/+^* alone did not lead to any visible tumors in our studies, indicating that LOH is required for *reck* tumorigenicity, or that *reck* is a weaker haploinsufficient TSG, compared to *tp53* or *rpL35* in zebrafish. TSGs usually function through LOH (two wildtype alleles are inactivated) or haploinsufficiency (one wildtype allele is inactivated) [37]. Half of our examined tumor samples (4 out 8) showed loss of the wildtype allele, suggesting that the *reck* gene may fit LOH, and it likely follows the two-hit paradigm of tumor suppression in zebrafish. However, we cannot exclude the possibility of the haploinsufficiency mechanism, since we were only able to examine 8 samples. Further studies with more tumors samples will be needed to address this question, and whether human RECK follows the same rule.

One of the interesting observations is that the tumor spectrum shifts in the *reck* mutant zebrafish. Generally, *tp53* or *rpL35* mutations lead to MPNSTs in zebrafish [32, 33]. In this study, 25% (6 out of 24) of tumors were pPNETs in *sdp^w12/+^;rpL35^hi258/+^*, while only 7% (2 out of 28) were pPNETs in *rpL35^hi258/+^*. A pPNET is a type of round cell sarcoma belonging to the Ewing family of tumors, which also originate from the neural ectoderm where neural crest cells develop [42]. Considering a potential developmental role of *reck* in the zebrafish neural crest (reported previously [34] and Supplementary Figure 1), the loss of *reck* function is likely responsible for the tumor cell differentiation to pPNETs instead of MPNSTs. RECK was recently found to regulate WNT [43, 44] and NOTCH [45] signaling pathways, which are important for neural crest differentiation [46, 47] and MPNST tumorigenesis [48–50]. Future studies are required to reveal the detailed mechanism behind this phenomenon.

Identification of new TSGs is not only important for the understanding of cancer biology, but also the key for cancer targeted therapy. Cancer targeted therapy is generally believed to be aimed at an oncogene. However, TSGs are also important for cancer targeted therapy, since the discovery of synthetic lethality of the *PARP1* and *BRCA1/2* genes and their clinical applications using PARP inhibitors [51, 52]. Along the same direction, TSG restoration was also demonstrated as another way of targeted therapy based on the biology of TSGs [53]. In the case of the *RECK* gene, it is frequently reported to be silenced through promoter methylation in a variety of human cancers [54–59]. Thus, attempts were made to restore RECK expression using DNA methyltransferase (DNMT) inhibitor, 5-aza-2′deoxycytidine (5-aza-dC), in colon cancer and salivary adenoid cystic carcinoma [60, 61]. Our cellular experiments on RECK restoration also support the possibility of RECK as a target for MPNST therapy. Furthermore, cancer driver genes that are located on the same chromosome region were found to cooperate and promote tumorigenesis [62]. The discovery of such combinations is very important in developing new cancer therapies. For example, this phenomenon has recently been reported in human colon cancers, where the *TP53* copy number loss was coupled with the loss of a neighboring gene, *POLR2A* [63]. Identification of other TSGs on the chromosome 9p might further help to understand the tumorigenic mechanism of RECK and to develop new cancer treatment strategies based on the concept of gene cooperation.

## MATERIALS AND METHODS

### Zebrafish lines, husbandry, and tumor onset analysis

Zebrafish were raised and maintained at the Purdue animal housing facility, which is approved by AAALAC. All experiments were carried out according to the protocols approved by the Purdue Animal Care and Use Committee (PACUC). The tumor-prone zebrafish lines carrying either the *tp53^M214K/M214K^* point mutation, or insertional mutations in ribosomal protein gene, *rpL35^hi258/+^,* have been described previously [32, 33]. The zebrafish *reck* point mutation carrier, *sdp^w12/+^,* was acquired from the laboratory of Dr. David W. Raible at the University of Washington. After crossing, genotyping was carried out at 6–8 weeks of age using previously published genotyping methods [13, 34].

After genotyping, siblings of different genotypes were housed in adjacent tanks at similar densities to minimize environmental differences. From the sixth month, we started to check for fish with visible tumors. During tumorigenesis, fish were euthanized at first observation of tumors or other signs of illness. Any accidental fish loss was censored. The tumor tissue samples from the euthanized fish were processed for hematoxylin and eosin (H&E) staining. The tumor types were confirmed by a board certified veterinary pathologist, blinded to the experimental groups. The final Kaplan–Meier tumor free survival curves were generated, and log-rank tests were performed to determine statistical significance using GraphPad Prism 6.0 h [13].

### RECK protein sequence and phylogeny analyses

RECK protein sequences were identified by a BLASTp search using the human RECK sequence as a query in Ensembl, NCBI, or JGI website. The longest sequence was preferentially chosen when there were multiple sequences. Multiple protein sequences (Supplementary Table 1) were aligned using MUSLE program [64], and the FASTA format alignment can be found in the Supplementary File 1. The evolutionary model for phylogenetic analysis was identified using a best model test using maximum likelihood, and default parameters in MEGA6 [65]. A maximum likelihood phylogenetic tree was constructed using JTT + G with 1000 bootstrap replicates with PhyML 3.1 [66]. The final phylogenetic trees were viewed and generated with FigTree V1.4.2 (http://tree.bio.ed.ac.uk/software/figtree). Gene intron-exon structures were analyzed using the longest transcripts in Ensembl.

### Cell culture, stable cell lines, and cell growth assays

All experimental protocols using cell lines and plasmid constructs were approved by Purdue University institutional review board. The human MPNST cell lines were authenticated by ATCC using short tandem repeat profiling (April 25, 2016). Cells were cultured in DMEM with 10% heat inactivated fetal bovine serum, penicillin (100 IU/ml), and streptomycin (100 μg/ml). All cell cultures were carried out at 37° C in a humidified 5% CO_2_ atmosphere. A full-length sequence of the *RECK* gene was amplified from HEK293T cells, and sequences were confirmed by Sanger sequencing. The *RECK* gene was subsequently cloned into pSlik-Neo vector (Addgene #25735). Stable cell establishment, MTT and plate colony assays were conducted following previously published methods [41]. For Western blot, commercial anti-RECK antibody (BD, #611512 or Cell Signaling #3433, 1:500 dilution) was used following an established method [41]. Statistical analyses were performed using GraphPad Prism 6.0 h. Data were analyzed using the un-paired student *t*-test. *p* < 0.05 was considered as statistically significant.

### Soft agar assay

For each cell line, 1000 cells per well were seeded in six-well plates. 1.5 ml 1% agarose was plated at the bottom of plate. After solidification, 1 ml of single-cell suspension in 0.7% agarose in DMEM supplemented with 10% FBS were added in each well. Then, the plates were incubated at 37° C in a humidified incubator with 5% CO_2_. On the second day, doxycycline (1 μg/ml) or PBS (phosphate-buffered saline) were added to induce RECK expression. The medium was refreshed every 2 days to maintain constant expression of RECK. Two weeks later, cells were stained with 0.01% crystal violet solution. After multiple washes with PBS, the cell colonies were visualized and imaged under a bright field microscope. Six sub-images at different focus in the same field were captured at 40× magnification, and all the images of the same field were Z stacked using Image J software. Ten random bright field images of each sample were captured. Total number of formed foci (diameter > 20 μm) in each field were counted manually in Image J software. Three technical replicates were included, and each experiment was repeated at least three times for each experimental condition.

### *In situ* hybridization

The primers Dr.reck-97F (5' CACCATGAGCGGGTGTCTCCAGATCCTCA3') and Dr.reck-2961R (5' GAGGTCAGAGGTCAGGGTCAGGAT3') were used for the zebrafish *reck* gene cloning PCR. The zebrafish *reck* gene cloning and *In Situ* hybridization were performed using the standard methods as previously described [67].

## SUPPLEMENTARY MATERIALS FIGURES AND TABLES





## Abbreviations

MPNST: malignant peripheral nerve sheath tumor
pPNET: peripheral primitive neuro-ectodermal tumor
TSG: tumor suppressor gene
CNAs: copy number alterations
LOH: loss of heterozygosity
