# Supplementary material for: Identification of RECK as an evolutionarily conserved tumor suppressor gene for zebrafish malignant peripheral nerve sheath tumors
Source: Oncotarget. 2018 May 4;9(34):23494–504. doi: 10.18632/oncotarget.25236 (PMC5955097; doi:10.18632/oncotarget.25236)
Supplement: Supplementary file 1 [file oncotarget-09-23494-s001.pdf]

Identification of *RECK* as an evolutionarily conserved tumor suppressor gene for zebrafish malignant peripheral nerve sheath tumors

SUPPLEMENTARY MATERIALS

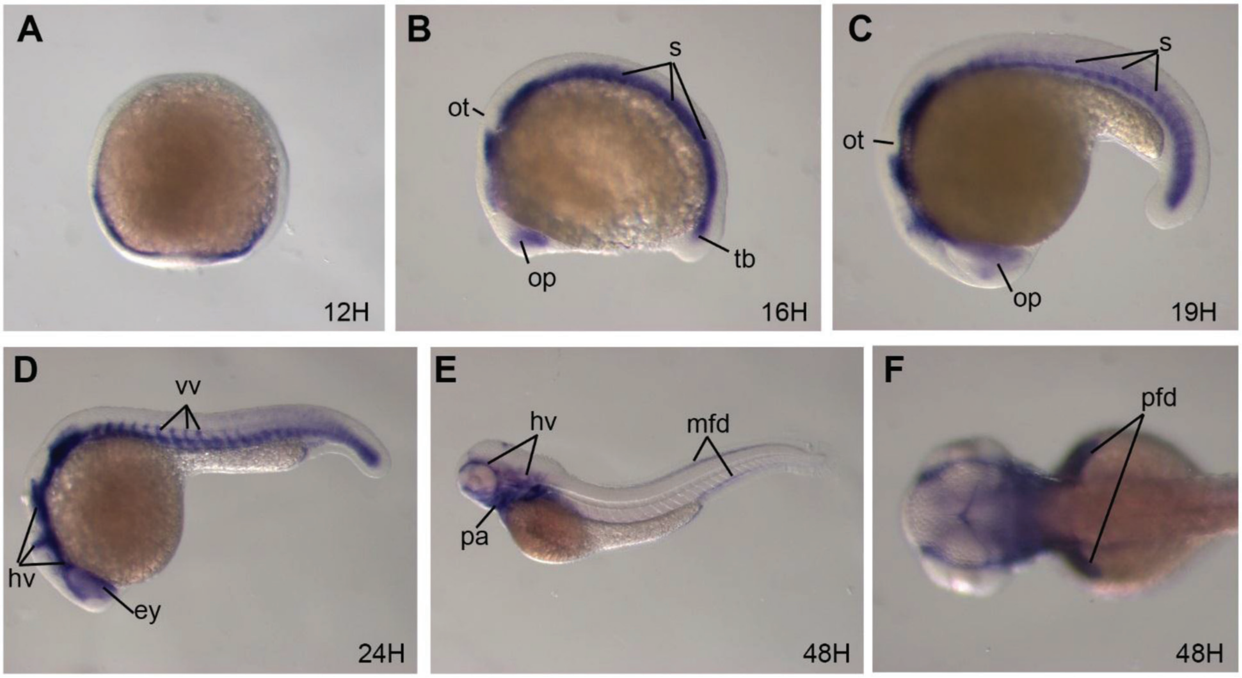

Supplementary Figure 1: The *reck* gene is expressed in neural crest derived and other tissue types during zebrafish embryogenesis. (**A**) *reck* is expressed in the head and tail region of the embryos at Stage 12 H (12 hours post fertilization). (**B**) At stage 16H, *reck* is expressed in the optic vesicle, head mesenchymal cells, and somite mesoderm. (**C**) At stage 19 H, the *reck* gene is expressed in a similar pattern with 16 H, but it is more evident in the ventral somites. (**D**) The *reck* expression is most evident in the head vasculature, eye, and ventral vascular primordia at the 24 H stage. (**E**) At stage 48 H, the *reck* gene is mainly expressed in the head vascular vessels, pharyngeal arches, and fin folds. (**F**) Higher magnification of the head region at stage 48 H (E). The *reck* expression is most evident in the head vasculature and pectoral fin fold. *ey*, *eye*; *hv*, head vesicles; *mfd*, middle fin fold; *op*, optic vesicle; *ot*, otic vesicle; *pa*, pharyngeal arches; *pfd*, pectoral fin fold; *s*, somite; *tb*, tail bud; *vv*, ventral vesicles.

Supplementary Table 1: RECK protein sequences used for constructing phylogeny

Common name	Scientific name	Sequence ID
**Alligator**	*Alligator mississippiensis*	XP_014458445.1
**Ant**	*Camponotus floridanus*	XP_011266337.1
**Barbel**	*Sinocyclocheilus rhinocerous*	XP_016394508
**Cavefish**	*Astyanax mexicanus*	XP_007233449.1
**Chicken**	*Gallus gallus*	RECK-201 ENSGALT00000020610
**Cod**	*Gadus morhua*	reck-201 ENSGMOT00000000165
**Coelacanth**	*Latimeria chalumnae*	RECK-201 ENSLACT00000008808
**Eagle**	*Haliaeetus leucocephalus*	XP_010575445.1
**Flour beetle**	*Tribolium castaneum*	XP_008197466.1
**Fopius**	*Fopius arisanus*	XP_011297418.1
**Fruit fly**	*Drosophila melanogaster*	Reck-RA FBtr0075671
**Fugu**	*Takifugu rubripes*	XP_011616177.1
**Honey bee**	*Apis mellifera*	NP_001242961.1
**Human**	*Homo sapiens*	RECK-001 ENST00000377966
**Japanese lamprey 1**	*Lethenteron japonicum*	JL7564
**Japanese lamprey 2**	*Lethenteron japonicum*	JL964
**Lancelet 1**	*Branchiostoma floridae*	JGI98411
**Lancelet 2**	*Branchiostoma floridae*	JGI215934
**Lizard**	*Anolis carolinensis*	RECK-201 ENSACAT00000009173
**Medaka**	*Oryzias latipes*	XP_004084760.2
**Mosquito**	*Anopheles sinensis*	KFB46496.1
**Mouse**	*Mus musculus*	Reck-001 ENSMUST00000030198
**Pea aphid**	*Acyrthosiphon pisum*	XP_001942581.2
**Placozoa**	*Trichoplax adhaerens*	XP_002110250.1
**Platyfish**	*Xiphophorus maculatus*	reck-201 ENSXMAT00000017382
**Rat**	*Rattus norvegicus*	Reck-202 ENSRNOT00000084031
**Sea lamprey**	*Petromyzon marinus*	reck (1 to many)-202 ENSPMAT00000001492
**Sea urchin**	*Strongylocentrotus purpuratus*	XP_790172.3
**Shark**	*Callorhinchus milii*	XP_007896317.1
**Spider**	*Parasteatoda tepidariorum*	XP_015925842.1
**Spotted gar**	*Lepisosteus oculatus*	XP_015210000.1
**Stickleback**	*Gasterosteus aculeatus*	reck-201 ENSGACT00000001249
**Tetradon 1**	*Tetraodon nigroviridis*	reck (1 to many)-201 ENSTNIT00000009626
**Tetradon 2**	*Tetraodon nigroviridis*	ENSTNIT00000009706
**Tilapia**	*Oreochromis niloticus*	reck-201 ENSONIT00000012127
**Turtle**	*Chrysemys picta bellii*	XP_005292683
**Xenopus**	*Xenopus tropicalis*	XP_002938983.2

Supplementary File 1: RECK protein sequence alignment. See Supplementary_File_1
