# Supplementary material for: Identification of RECK as an evolutionarily conserved tumor suppressor gene for zebrafish malignant peripheral nerve sheath tumors
Source: Oncotarget. 2018 May 4;9(34):23494–504. doi: 10.18632/oncotarget.25236 (PMC5955097; doi:10.18632/oncotarget.25236)
Supplement: Supplementary file 2 [file oncotarget-09-23494-s002.docx]

**Supplementary File 1. RECK protein sequence alignment.**

10 20 30 40 50 60 70 80 90 100

....|....|....|....|....|....|....|....|....|....|....|....|....|....|....|....|....|....|....|....|

**Mouse**  **----------------------------------------------------------------MASVRASPRSALLL-----------LLAAAGVAEVT**

**JPlamprey 2** **----------------------------------------------------------------------------------------------------**

**JPlamprey 1** **----------------------------------------------------------------------------------------------------**

**Tetradon 2**  **----------------------------------------------------------------------------------------------------**

**Bf98411**  **----------------------------------------------------------------------------------------------------**

**Stickleback** **----------------------------------------------------------------------------------------------------**

**Bf215943**  **----------------------------------------------------------------------------------------------------**

**Aphid**  **------------------------------------------------------MIRHTPDHGIIMFIVYFLLVLCAG----------------------**

**Turtle**  **----------------------------------------------------------------------MELTAPPR-----------PWAFLWLAAA-**

**Fruit fly**  **MRLSGWEILLFLLPFGVVSAVRQLEENQSIKNDHHHSVERQVRRRNNKSNASVDHHRHAPSSGSKKNSRSHSVSLDSDDYDWLDADTSEAIDSSEVTAVE**

**Honybee**  **----------------------------------------------------------------------------------------------------**

**Ant**  **-------------------------------------MVAEGFKGISQVHTDKMHYLTMLLITTIAILFAPVVANP------------------------**

**Tetra**  **------------------------------------------------------------------------------------MCARSQIVFLLLACNF**

**Sea urchin**  **----------------------------------------------------------------------------------------------------**

**Mosquito**  **------------------------------------------------------MVSHATTATDRPASRWCIVPQQ-----------------------R**

**Cavefish**  **------------------------------------------------------------------------------------MCARSQIVFLLLACNF**

**Rat**  **----------------------------------------------------------------MASVRASARGALLL-----------LLSAAGVAKVT**

**Medaka**  **----------------------------------------------------------------------------------------------MTHILT**

**Human**  **----------------------------------------------------------------MATVRASLRGALLL-----------LLAVAGVAEVA**

**Tilapia**  **-----------------------------------------------------------------------------------MRLCFQITSFLLAVNFY**

**Lizard**  **-------------------------------------------------------------MAARAGPPQQGTLETRM-----------ALLAAPALLLL**

**Shark**  **----------------------------------------------------------------------MAQSSIFA--------------LLLLVMVL**

**Fugu**  **-----------------------------------------------------------------------------------------------MNDGS**

**Coelacanth**  **---------------------------------------------------------------------MFGYMEMIS--------------LFLLLHLC**

**Spotted gar** **-------------------------------------------------------------------------------------MFVCFEMIIFFLVFN**

**Fopius**  **-------------------------------------------MRDSTMWTVGFIFLHLWIFGDTRNSLADIS---------------------------**

**Beetle**  **--------------------------------------------------------MSFQTNKSAIFSLCFIVLYT------------------------**

**Barbel**  **------------------------------------------------------------------------------------MYVCCEIIILLLSCNF**

**Platyfish**  **----------------------------------------------------------------------------------------------------**

**Zebrafish**  **------------------------------------------------------------------------------------MSGCLQILTVLLCCRF**

**Xenopus**  **-------------------------------------------------------MRRRSGDVMHAAKRVMSASASCLGLLGIWLFLIVFFILEAAGTRS**

**Chicken**  **----------------------------------------------------------------------MAAAVAAW-----------PWALFCLAAVP**

**Aligator**  **----------------------------------------------------------------------------------------------------**

**Eagle**  **--------------------------------------------------------------------------------------------MWLEAEGR**

**Spider**  **----------------------------------------------------------------------------MREKSWHSTCYGHRILTAIIVIGL**

**Placozoa**  **----------------------------------------------------------------------------------------------------**

**Cod**  **----------------------------------------------------------------------------------------------------**

**Sea Lamprey** **----------------------------------------------------------------------------------------------------**

**Tetradon 1**  **----------------------------------------------------------------------------------------------------**

110 120 130 140 150 160 170 180 190 200

....|....|....|....|....|....|....|....|....|....|....|....|....|....|....|....|....|....|....|....|

**Mouse**  **GGLAPGSAGAVCCNHSKDNQMCRDVCEQIFSSKSES---RLKHLLQRAPDYCPET-MVEIWSCMNSSLPGVFKKSDGWVGLGCCELAIGLECRQACKQAS**

**JPlamprey 2** **----------------------------------------------------------------------------------------------------**

**JPlamprey 1** **----------------------------------------------------------------------------------------------------**

**Tetradon 2**  **----------------------------------------------------------------------------------------------------**

**Bf98411**  **---------------------------------------------------MLLS-GAGVPIMPCCLRTGEPESGGEWVGHACCDLTVSDLCKTACLEAS**

**Stickleback** **---------------------------QLATIKSES---HLNHLLQRLPSYCPEL-MNELWTCINSSLPGVSRKSEGWVGLGCCELSISPECRRECRQAS**

**Bf215943**  **-----------------------------------------------------------------------------WVGHACCDLTVSDLCKTACLEAS**

**Aphid**  **----RLIDAEKAKCCSRVTGNCRRACEQLAVLQEYP-KDKIAGRITDFYNQCSSKNVRHVEWCMSSSAQ-DTEIKWLDAARKCCDVARSDSCQLSCLDQN**

**Turtle**  **ALLLPGAAGMSCCNHAKDNLMCRDVCEQILSSKSDS---RLKHLLQRAPEYCPES-MGEVWGCINSSLPGVLKKSDGWVGLGCCELAIVVECRQACKQAS**

**Fruit fly**  **SIRSPGESYDIFTCCNQVFGSCRTACENLSLVEFATGTGG--DNRDELHRYCQLH-QVEFWTCVNQTFD-AITRGADWSGRRCCQFGVLPHCRNVCATST**

**Honybee**  **-----------MSCCSLAAGSCRNDCSKISLVALGAEAEARENATQRLLELCSLE-LTEFWGCVNSTLN-EVKRHENWTGRRCCYLVQNPICRSTCALSG**

**Ant**  **----FFEAAQEMSCCSLAAGSCRNVCSKISLVVLGAEIEARENANRRLLEFCPME-LMDFWFCVNSTLN-ELKRNENWIGRGCCRLIQNPTCRTTCALSG**

**Tetra**  **CALVYAQDPSCCHH-AAEFPQCREACDQLAKIRSDS---HLRFLYQRLSSYCSES-MTELWICINSTLPGASRKSYGWVGLGCCELAISAECRRECKQAS**

**Sea urchin**  **---------------------------MVLESTTAG---RRKHMGT-LLNACPST-LNSFWRCLNNSASDIWQQDTEWPGHWCCPNAISGHCRVACSQAH**

**Mosquito**  **PGRGQQRYTDGFTCCTEVQGSCKAACENLSLARIASEPGSREAKLADVRKYCPLQQQAPFWQCMNETLA-AIQRGAAWPGRVCCPLAMSVRCQNVCATGS**

**Cavefish**  **CALVYAQDPSCCHH-AAEFPQCREACDQLAKIRSDS---HLRFLYQRLSSYCSES-MTELWICINSTLPGASRKSYGWVGLGCCELAISAECRRECKQAS**

**Rat**  **GGLAPGSAGAVCCNHSKDNQMCRDVCEQIFSSKSES---RLKHLLQRAPDYCPET-MVEIWSCMNSSLPGVFKKSDGWVGLGCCELAIGLECRQACKQAS**

**Medaka**  **DINTPVTNQTPHDKGAAEEDLGSVLFLQLASIKSES---HLKHFLQRLPSFCPES-MSDLWVCINSSLPGVSRKSGGWVGFGCCELAISTECRSKCQHAS**

**Human**  **GGLAPGSAGALCCNHSKDNQMCRDVCEQIFSSKSES---RLKHLLQRAPDYCPET-MVEIWNCMNSSLPGVFKKSDGWVGLGCCELAIALECRQACKQAS**

**Tilapia**  **SRPVRAQ-DPSCCHHAAEFSPCREACDQLTTIKSES---RLKHLLQRLPGYCSES-MNELWMCINSTLPGVSRKSEGWVGLGCCELAITTECRKECKQAS**

**Lizard**  **LLWLPGLADTSCCYLAKGNLMCRDVCEQILTSKSDS---HLRHLLQRAPEYCPES-MGEVWGCINSSLLGVLKKSDGWVGLGCCELAIVVECRQACKQAS**

**Shark**  **KIFRCGAEDVSCCYQAREYPICRDACEQLTSTKSES---RLKHLLQRLPGYCPES-MVEFWVCINSSLPGASKKPEGWVGLGCCELAIAVECRRACKQAS**

**Fugu**  **SSPVLAWTDPSCCHQAAEFTPCKEACDQLATIKSES---RLKHLLQRLPGYCPES-MNELWTCINSTLPGVSRKSEGWVGLGCCELAISAECRRECRQAS**

**Coelacanth**  **SPAQS--QDASCCYHARDNPMCRDACEQLTATKSES---RLKYLLQRLPEYCPQS-MVDFWVCINSTLPGVSKKSDGWVGLGCCELAISTDCRRSCKQAS**

**Spotted gar** **IHDFVQAQDSLCCHHGREISRCREACEQLSTIKSES---RLKHLLQRLPSYCPES-MNELWVCINSTLPGASKKSEGWVGLGCCELAIDPDCRQECKLAS**

**Fopius**  **----------EMSCCSYATGSCRSVCSQISLVALGADPIARENSSRQLQEFCSME-LTQFWTCVNSTLD-EARKNEDWSGRQCCQLAQYPICQSTCALAG**

**Beetle**  **-----RILAQDLMCCSHTTGSCRSVCEKISLAQLAADSRLRNETVEEVRKFCSPQ-LSPFWECLNATFK-DMSRGESWSGRVCCPIPQSNSCRKACITAT**

**Barbel**  **CALVYTQDPSCCHHAAELFPQCRGACEQLATIRSES---RLKHLLLRLPSYCPES-LSELWICINNTLPGASKKSDGWVGLGCCELAISAECRRDCKQAS**

**Platyfish**  **---------------------------QLATIKSES---RLKHLLQRLPNYCPES-LNELWMCINSTLPGVSKKSDGWVGLGCCELAIAGECRRECKQAS**

**Zebrafish**  **WALVFSQDQSCCVHHAADIPRCRDACEQLASIRSES---RLRHLLHRLPSYCPET-LSELWICINNSLPGASRKSDGWVGLGCCELAISAECRRDCKQAS**

**Xenopus**  **SAFSSMASDASCCNQAKDNLMCRDVCEQILSSKSES---RIKHLFLRAPDYCPTS-MIDVWTCINSSLPGVSKKSDGWVGLGCCELAIAVECRRACKQAS**

**Chicken**  **PLLSPGAAGLSCCYHAKDNLMCRDVCEQILSSKSDS---RLKHLLQRAPEYCPES-MGEVWGCINSSLPGVLKKSDGWVGLGCCELAIAVECRQACKQAS**

**Aligator**  **---------MSCCYLAKDNLMCRDVCEQILSSKSDS---RLKHLLQRAPEYCPES-MGEVWGCINSSLPGVLKKSDGWVGLGCCELAIAVECRQACKQAS**

**Eagle**  **GVRSPFGGSLSCCYHAKDNLMCRDVCEQILSSKSDS---RLKHLLQRAPEYCPES-MGEVWGCINSSLPGVLKKSDGWVGLGCCELAIAVECRQACKQAS**

**Spider**  **LDISWMAHAASLRCCSHANGSCRSACEQISLVALATDQRELEINLQKLNDLCPKSPMLAFWNCMNESLAEIDKGS-GWIGRPCCGIPQSEKCKLACLQAQ**

**Placozoa**  **-----MLLLPCFILATAFLSSSLITATTTSCCNLATDSLDCKAACLELYGVHPGD-MEGYTKSVTALLERCPSNNFKLNNGDICSHYVGRINVFDVRFGN**

**Cod**  **---------------------------QLGTIKSES---RLKHLLLRLPGYCPVS-MXXXXXXXXXXXXXVSRRSDGWVGLGCCELAMSVECRRECKQAA**

**Sea Lamprey** **--------------------------------------------------------------------PGVQRKPEGWVGLGCCELAIANECRRACRQAS**

**Tetradon 1**  **-----------CCHHAAEFTPCKEACDQLATIKSES---RLKHLLQRLPGYCPES-MNDLWTCINSTLPGVSRKSEGWVGLGCCELAISAECRRECRQAS**

210 220 230 240 250 260 270 280 290 300

....|....|....|....|....|....|....|....|....|....|....|....|....|....|....|....|....|....|....|....|

**Mouse**  **SKNDI-SKVCRKEYENALFSCISRN-EMGSVCCSYAGHHTNCREFCQAIFRTDSSPGPSQIKAVENYCASISP--------------QLIHCVNNYT-QS**

**JPlamprey 2** **----------------------------------------------------------------------------------------------------**

**JPlamprey 1** **-------LRARACVCVSLPSHAGRR-AVGSLCCGHAGRHTNCREFCTAVFRSDAPPTPGQIRSVESYCATVSV--------------AVVDCVDNYT-RS**

**Tetradon 2**  **----------------------------------------------------------------------------------------------------**

**Bf98411**  **TSSDISAISCRRDLEPALHSCIERN-EMGERCCGTS-VSTECEEACSTVFQTDSTPTRQQRQAIQDHCMLDNQ--------------QVIQCVDNYT-NS**

**Stickleback** **SKNDI-TKVCKKVTENSLYSCITKNEKVGSTCCSYAGRHTTCREYCQAIFRTDSTPTVSQISAVKEYCQSHSP--------------QLLSCVTNFT-KS**

**Bf215943**  **TSSDISAISCRRDLEPALHSCIERN-EMGERCCGTS-VSTECEEACSTVFQTDSTPTRQQRQAIQDHCMLDNQ--------------QVIQCVDNYT-NS**

**Aphid**  **SESRS-VHKN-CQDETDFFNCLQHQ-QVKDTCCGDKTDAVQCQIACEKFLANTPSINRHAANQEILDLCSADRRDARSATTATTSVELHRRCTRNTT--A**

**Turtle**  **SKNDI-LKVCRKEYENALFSCINRN-EMGSVCCSYAGHHTNCREYCQAIFRTDSSPGPSQIKAVESYCASISP--------------QLIHCVNNYT-QS**

**Fruit fly**  **RSP---VQNCRRSDEQTLYDCLERQ-EAADQCCGQARTS-ECLEACRAVFE-PAN--DNHDHVDINGACGDRN-------------ADVIQCVNNHT--D**

**Honybee**  **SRRNL-NESCRPSDEPEFFSCLERR-EEAEHCCGKVSND-TCRAVCQNLFY-KREKIS-----SRNGCFH-----------------QMPKCLKSVT--E**

**Ant**  **STNDL-NESCRPSDESEFFFCLEKR-EEAERCCSNVSND-TCRSICKDIFY-KPSKQSSLKLYSSKGCFH-----------------QIPKCLKSVA--E**

**Tetra**  **SKNDI-TKICKKDTEKPLYSCITKN-EMGSVCCSYAEKQTNCREYCQAIFRTDSSPTSSQISAVKQYCQGTSP--------------QLIGCVANYT-KS**

**Sea urchin**  **SFSEV-AAACTHSEEGDLFGCLDRY-ERRESCCSLAHEGTQCRNKCEDVFDS-PLPTDNMGLDIEIYCSDER----------------IKQCVYNYTNIM**

**Mosquito**  **SRDEL-VAGCRASDEQSLFGCLKRQ-EDGHRCCSSARTS-ECVHACSEVFQ-RYRTPSKAQREEVLAACRTNN-------------SNVMQCIKGFI--D**

**Cavefish**  **SKNDI-TKICKKDTEKPLYSCITKN-EMGSVCCSYAEKQTNCREYCQAIFRTDSSPTSSQISAVKQYCQGTSP--------------QLIGCVANYT-KS**

**Rat**  **SKNDI-SKVCRKEYENALFSCISRN-EMGSVCCSYAGHHTNCREFCQAIFRTDSSPGPSQIKAVENYCASISP--------------QLIHCVNNYT-QS**

**Medaka**  **SRNDI-TEVCKKVTEVSVCACVCVR-TEGSSCCVHAGRHTICRDYCEAIFRTDSTPSRSQIQAVTGYCQSHST--------------KLVDCVGNFT-ES**

**Human**  **SKNDI-SKVCRKEYENALFSCISRN-EMGSVCCSYAGHHTNCREYCQAIFRTDSSPGPSQIKAVENYCASISP--------------QLIHCVNNYT-QS**

**Tilapia**  **SKNDI-TKVCKKTTENSLYSCITKN-EMGSTCCSYAGRHTTCREYCQAIFRTDSTPTVSQISAVKDYCQTHSA--------------QLIHCVNNFT-KS**

**Lizard**  **SKNGI-LKICRKEYENALFNCINRN-EMGSVCCSYAGHHTNCREYCQAIFRTDSSPGPSQIKAVESYCASVSP--------------QLIHCVNNYT-QS**

**Shark**  **SKND--LKSCRKEYETAFYSCVNRN-EMGSLCCSYAGRHTNCREYCQAIFRTDSSPSQSQIRAVENYCASISP--------------QLIHCVDNYT-RS**

**Fugu**  **SENDI-IKVCKKVTENSLYSCITKN-EMGSTCCSYAGRHTTCREYCQAIFRTDSTPTMSQINAVKEYCQSHSA--------------QLLTCVSNFT-KS**

**Coelacanth**  **SKNDI-LKSCKKEHETALFTCVNRN-EMGSVCCSYAGKHTNCQEYCRAIFRTDSSPTPSQIKAVKNYCAAISP--------------QLMECVDNYT-ES**

**Spotted gar** **SKNDI-RKKCEKGFENALYSCISRN-EMGSVCCSYAGRHTNCREYCQAIFRTDSPPTLSQIKAVEDYCQSISP--------------ELISCVENYT-ES**

**Fopius**  **SRESL-RGSCRPSDEPEFYTCLERR-EDGEKCCSNVSNA-TCRAVCRELFD-NPKKPSSLKVYSSKGCFH-----------------QVPKCLKNVA--D**

**Beetle**  **STQDL-AQGCRQSDEIEFFRCLERQ-KSGEECCSNARSD-DCHQVCLEIFR-SRWTPNPPLRLKVQEMCEANS-------------PKVMDCVKDFI--K**

**Barbel**  **SKNDI-AKVCKKDTEKPLYSCITKN-ESKQSCMTRS--------------------------------LVISP--------------SLIGCVANYT-KS**

**Platyfish**  **SKNDI-TKVCKKVTENSLYSCITKN-EMGSACCGFVGRHTNCRDFCDAIFRTDSTPTESQIKTVKDFCKSHSP--------------KLVECVNNFT-ES**

**Zebrafish**  **SKNDI-SKVCKKDTENPLYSCITKN-EMGSVCCSYAGRHTTCREYCQAIFRTDSSPTVSQISAVKEYCQSVSP--------------PLILCVENYT-RL**

**Xenopus**  **SKNDI-SKACRKQYETALISCINRN-EMGSVCCSYAGRHTNCREYCQAIFRTDSSPGPSQIKAVENFCASISP--------------PLVQCVNNYT-QS**

**Chicken**  **SKNDI-LKVCRKEYENALFSCINRN-EMGSICCSYAGHHTNCREYCQAIFRTDSSPGPSQIKAVENYCASISP--------------QLIHCVNNYT-QS**

**Aligator**  **SKNDI-LKICRKEYENALFSCINRN-EMGSVCCSYAGHHTNCREYCQAIFRTDSSPGPSQIKAVENYCAAISP--------------QLIHCVNNYT-QS**

**Eagle**  **AKNDV-LKVCRKEYENALFSCINRN-EMGSVCCSYAGHHTNCREYCQAIFRTDSSPGPSQIKAVENYCASISP--------------QLIHCVNNYT-QS**

**Spider**  **SVLDL-TIACRKSDEIAFYTCIEKQ-EAGERCCKNA-QTENCLEACKNVFSDAFTPTKVVRDAYHKHCSEGNP--------------RIQQCIHTYI-SV**

**Placozoa**  **KSTTFEVYPYDSINISSHMLHQQNK--------TWKTQFWQCIPPTRSEFFVGTPALFDDQPIWRDCCNVTVTN------------RCRKTCLKHFLMAC**

**Cod**  **SKNDI-TKICKKDAEXXXXXXXXXX-XVGSTCCSYAGRHTTCREYCQAIFRTDSSPTVSQINAVKDYCQSHST--------------QLIGCVGNYT-KS**

**Sea Lamprey** **AKSGI-SSQCRKEYETALYNCVNRN-EMGSLCCSHAGRHTNCREYCQAIFRTDSAPTPNQIRTVENYCSVASP--------------AVVRCFDNYT-QS**

**Tetradon 1**  **SRSDI-IKVCKKLTENSLYSCITKN-EMGSTCCSYAGRHTTCREYCQAIFRTDSTPTSSQITAVTEYCQSHSA--------------QLLACVANFT-KS**

310 320 330 340 350 360 370 380 390 400

....|....|....|....|....|....|....|....|....|....|....|....|....|....|....|....|....|....|....|....|

**Mouse**  **YPMRNPTDS-LYCCDRAEDHACQNACKRILMSKKT-------EMEIVDGLIEGCKTQPLPQDPLWQCFLES-------------------SQSVHPGVTV**

**JPlamprey 2** **---------------------------------------------MVEGLIEGCGRQPLPQDPLWQCFLEGADTG-----------GGRRRAGGGGGAQD**

**JPlamprey 1** **YPSRNPVDSGLHCCERAGDAACEASCRSVLHTMAS-------EQEMVEGLIAGCGRQPLPQDPLWQCFLEGAHVEPG----AGAGPGGSRRAG--GAVSD**

**Tetradon 2**  **----------------------------------------------------------------------------------------------------**

**Bf98411**  **RPANNPAES-LHCCDQSNDEQCQDRCRRVLVTLTS-------EQEIVDGLIEGCGD-PLPQDPLWQCFLS--------------------ASANNGHDTE**

**Stickleback** **YPVRSPIDS-LHCCDRAEATHCQVACRRILRTMST-------EHEIMEGLINECGSQPLPQDPMWQCFLG--------------------SAHPASPPEV**

**Bf215943**  **RPANNPAES-LHCCDQSNDEQCQDRCRRVLVTLTS-------EQEIVDGLIEGCGD-PLPQDPLWQCFLS--------------------ASANNGHDTE**

**Aphid**  **RRPSSDGHKYLHCCKLAADTQCEQACQDSLRRKFD-----SDVEAVDSLEGR-GCGSPSLDDRFWQCFLQSE--------RAEQKDTSAP-------APM**

**Turtle**  **YPMRNPTDS-LYCCDRAEDYTCQSACKKILMSKKT-------ELEIVDGLIEGCKTMPLPQDPLWQCFLES-------------------SRSVHPGVTV**

**Fruit fly**  **MTPLANAEQYIPCCEYSSKEHCRHTCRNLLHQNATLME--RSEVIFGRLEAAG-CGSPLPQLPFWQCFLTVTGKLYVPGGSGRAGGSLAQGRISGQGGSL**

**Honybee**  **VKHAENPKQHLHCCNKADSLTCMETCKKILYTAT------TDREILNALTEK-CGPPISPQSPFWSCLMKSG---------------------------S**

**Ant**  **VKHAEDPKQHLHCCEEATTPACLDTCRKTLHIAN------TDQEIMDALAEK-CSP-VLPQSPMWSCLLKSG---------------------------S**

**Tetra**  **YPTRSPIDS-LYCCDRAEEAHCQTACKRILRTMTT-------EHEIMEGLINECGSQPLPQDPLWQCFLG--------------------SAHPPAKSDP**

**Sea urchin**  **APRHNPRDS-LHCCERSTRPRCREVCRRTLTNLTS-------EGAIMNALEEGC-DQPVPNDAMWRCFLLVS------------------AGNEISHPEI**

**Mosquito**  **VTFTPNLKRYVPCCDSSPEPKCRKVCRETLTAGGPDSEPYTVEEIIDLLQHRGGCGALMPTLPLWKCFIEAD--------KQRTAGQQVN------SNEV**

**Cavefish**  **YPTRSPIDS-LYCCDRAEEAHCQTACKRILRTMTT-------EHEIMEGLINECGSQPLPQDPLWQCFLG--------------------SAHPPAKSDP**

**Rat**  **YPMRNPTDS-LYCCDRAEEHACQSACRRILTSKKT-------EMEIVDGLIEGCKTQPLPQDPLWQCFLES-------------------SQSVHPGVTV**

**Medaka**  **YPVYSPTES-LYCCERADSTHCQLACRQILSTMTT-------EHEIIDGLIKECSS-PLPQDPMWQCFLG--------------------SALPPLPPEE**

**Human**  **YPMRNPTDS-LYCCDRAEDHACQNACKRILMSKKT-------EMEIVDGLIEGCKTQPLPQDPLWQCFLES-------------------SQSVHPGVTV**

**Tilapia**  **YPIHSPVDS-LYCCDR-AATHCQMACRQILRTMST-------EHEIMEGLIKECGSQPLPQEPMWQCFLS--------------------SVQTPS-ITP**

**Lizard**  **YPMRNSTDS-LFCCDRAEDYACQTACKRILMSMKT-------ELEIVDALIDSCKTMPLPQDPLWQCFLES-------------------SRSVHPGVTL**

**Shark**  **YPMKNPIDS-LYCCDRAEDHACQSTCRRVLMTMAT-------EHEIVDGLIEGCKKMPLPQDPLWQCFLES-------------------SGSNNT---S**

**Fugu**  **YPIRSPLDS-LHCCDRAEATHCQVACRRILRTMNT-------EHEIMEGLIEECGSQPLPQDPMWQCFLG--------------------SAHPPSPPKV**

**Coelacanth**  **YPMRKPVDS-LYCCDRAKDFACQTACKRILMTMTT-------ELEIVDGLIEGCKTKPLPQDPLWKCFLIN-------------------PVVPDP---D**

**Spotted gar** **YPIKNPIDS-LYCCERADDAHCQTACKRILRTMKT-------ENEIIDGLIKECDKQPLPQDPLWQCFLGN-------------------AGSGNNGVSV**

**Fopius**  **APNAENPKQYLQCCHEAPNAPCLDACRHVLLTAV------TIKDILDTLEEK-CPP-VQPHSPFWSCFLQAS---------------------------P**

**Beetle**  **VTPAKDLHKKKHCCDMSNQSKCRETCKKLLSMKN-----ITVQESIDGLQLGG-CGSPLPQEYFWSCFLKST----------ETAETTVE---------V**

**Barbel**  **YPIRSPIDS-LYCCDRAEEAHCQTACKRILRTMST-------EQEIMEGLISECGSQPLPQDPLWQCFLG--------------------SAHPPTISDP**

**Platyfish**  **YPARSPIDS-LYCCDRAEAPNCQTACRHLLRTMNT-------DQEIMEGLIKECGTQPLPQDPMWQCFLS--------------------RAHPPSPPEE**

**Zebrafish**  **HPTHRPIDS-LHCCDRAEEAHCQLACKRILRTLST-------EQEIMDGLISECGSQPLPQDPLWQCFLG--------------------SAHPPANTDP**

**Xenopus**  **YPMRNPVDS-LYCCDRAEDPRCQAACKRILMSKKT-------EPEIVDSLSEGC-TKPLPQDPLWQCFLES-------------------SRAVRPGVNI**

**Chicken**  **YPMRNPTDS-LYCCDRAEDYACQTACKRILMSMKT-------ELEIVDGLIEGCKTMPLPQDPLWQCFLES-------------------SRSVHPGVTV**

**Aligator**  **YPMRNPTDS-LYCCDRAEDYGCQTACKRILMSMKT-------ELEIVDGLIEGCKTMPLPQDPLWQCFLES-------------------SRSVHPGVTV**

**Eagle**  **YPMRNPTDS-LYCCDRAEDYACQTACKRILMSMKT-------ELEIVDGLIEGCKTMPLPQDPLWQCFLES-------------------SRSVHPGVTV**

**Spider**  **TPADNPFKN-MHCCEKSPNLRCRTVCKEALRTETT-------DQDIIDVLSRSECGNPLPQDRLWQCFLIN-------------------SAANEPGGSL**

**Placozoa**  **IERKKVSQG--CCNIASIGSRCSRACNMIFRNDIN---------ISRAHLSDLKYHCKYADPEVYRCAMEYAQS----------------DLAKQTVPGL**

**Cod**  **YPIRNPIDS-LYCCDRAEDAHCQVACRRILRTLST-------EQEIMEGLIEQCRSQPLPQDPMWQCFLG--------------------SAQPPAKPHE**

**Sea Lamprey** **YPARNPLDS-LHCCERAADSKCEEACRRVLVTIAS-------EQEMVEGLIEGCGRQPLPQDPLWQCFLEGADTG-----------GGRRRAGGGGGAQD**

**Tetradon 1**  **YPVRSPIDS-LHCCDRAEATHCQVACRRILRTMNT-------EHEIMEGLIQECGSQPLPQDPMWQCFLG--------------------SAHPPSPPEE**

410 420 430 440 450 460 470 480 490 500

....|....|....|....|....|....|....|....|....|....|....|....|....|....|....|....|....|....|....|....|

**Mouse**  **HPPPSTGLDGAKLHCCSKANTSTCRE-------------------LCTKLYSMSWGNTQSWQEFDRICEYNPVEVSMLTCLADVREPCQLGCTNLTYCTN**

**JPlamprey 2** **AASSPTGLDGAKLQCCSRANSPHCRW----------------LRDLCVGLFSMSWGSAQSWQEFERECEYRLQEPSMMGCLADVREPCQLGCRDLNYCTN**

**JPlamprey 1** **VGSSPTGLDGAKLQCCSRATSPLCRCSRSSTQPGGSLRHGPEVRELCVGLFSTSWGSAQRWQEFERECEYREQEAPMKGCLADVHEPCQLGCKDLSYCTN**

**Tetradon 2**  **----------------------------------------------------------------------------------------------------**

**Bf98411**  **EVIPVTGLDGAKLQCCSKAATSRCRD-------------------LCVKLYSTTWATSSSWNQFYADCQYHPSEEVLMQCIADVQEPCQLGCSELNYCTN**

**Stickleback** **ETPHPAKMDCAKLHCCAKANTSMCRD-------------------TCQEIS-TNWG-SQTGQDFDQFCEYNPVETELINCLADVREPCQLGCKDLTYCTN**

**Bf215943**  **EVIPVTGLDGAKLQCCSKAATSRCRD-------------------LCVKLYSTTWATSSSWNQFYADCQYHPSEEVLMQCIADVQEPCQLGCSELNYCTN**

**Aphid**  **SQIEKLGMDSVKLHCCDKATIAGCRK-------------------LCLKTFTNEWSS--SWNDFNSECLSHPNEDQLLKCLDDVEEPCDLGCDGLNYCTV**

**Turtle**  **HPPPSTGLDGAKLHCCSKANTSTCRE-------------------LCTKLYSTSWGNTQSWQEFDRICEYSPVEVSMLTCLADVREPCQLGCRNLTYCTN**

**Fruit fly**  **AEPNHLGMDAAKRQCCEQASSHKCRR-------------------LCNQIFTSNWWD--ARSSFENECMDQPGERELRRCIESVDAPCELGCQGLSFCSN**

**Honybee**  **PKPVRLPLDAGKLSCCTKAIKPKCQD-------------------LCWRAFKADWES--SWHPLDAECLSSSQEGELRRCLEDTDDPCEMGCAGLSYCTR**

**Ant**  **TKPARLPLDAGKLACCTKATRPTCQN-------------------LCWRAFQADWES--AWAQLDALCLSSSLEGELRRCLEDADDPCEMGCSGLSYCAR**

**Tetra**  **ETAPTAKMDCAKLHCCSKANTSLCRK-------------------MCQEIS-TNWG-TQTWQEFDQLCEYNPVETDLITCLADVREPCQLGCKDLTYCTN**

**Sea urchin**  **GPTNPGALDGANLQCCLRAISSRCRD-------------------ICVKLYTPSLSNRDTWNEFDEHCRYQPAEEALLTCLADVEEPCKAGCSGLDYCAH**

**Mosquito**  **SRINQIGMDSAKQHCCMKAESLRCRL-------------------LCSTTFANEWT----LDDLEQECLGAPEEHALRQCIDEVDEPCELGCDGLSFCSN**

**Cavefish**  **ETAPTAKMDCAKLHCCSKANTSLCRK-------------------MCQEIS-TNWG-TQTWQEFDQLCEYNPVETDLITCLADVREPCQLGCKDLTYCTN**

**Rat**  **HPPPSTGLDGAKLHCCSKANTSVCRE-------------------LCTKLYSMSWGNTQSWQEFDRICEYNPGEVSMLACLADVREPCQLGCTNLTYCTN**

**Medaka**  **ETPHPAKMDGAKLHCCSKANSSLCRD-------------------MCQKIS-LHWD-SQTWQDFEQFCEYKAAETELISCLSDVREPCQLGCKDLTYCTN**

**Human**  **HPPPSTGLDGAKLHCCSKANTSTCRE-------------------LCTKLYSMSWGNTQSWQEFDRFCEYNPVEVSMLTCLADVREPCQLGCRNLTYCTN**

**Tilapia**  **EDNLPAKMDCAKLHCCSKANTSLCRD-------------------MCLEIS-SNWG-SQTWQDFDQLCEYNPVETELINCLADVREPCQLGCKDLTYCTN**

**Lizard**  **HPPPSTGLDGAKLHCCSKANTSMCRD-------------------LCTKLYSSSWGNTQTWQEFDRFCEYNPAEVSMLTCLADVREPCQLGCRNLTYCTN**

**Shark**  **NGLPATAVDGAKLHCCSKANTSVCRD-------------------LCIKLYSTRWGSATHWQEFDRICEYNPQEASMFMCLADVREPCQLGCQSLKYCTN**

**Fugu**  **ETPHPSKMDCAKLHCCSKANTSLCRD-------------------MCQEIS-TNWG-SQTWQDFDQLCEYNPVEAELLNCLADVREPCQLGCRDLTYCTN**

**Coelacanth**  **VIHPPTGIDGAKLQCCSKANTSVCRE-------------------LCQKLYSTSWGNRQSWQEFDRICEYNPVESSMLTCLADVREPCQLGCKDLTFCTN**

**Spotted gar** **DTSPSTGLDSAKLHCCYKANTTICRE-------------------LCFTVS-TNWGSTQSWQEFDQFCEYNPMEAAMVNCLADVREPCQLGCKDLTYCTN**

**Fopius**  **IKPSRLPLDIGKLGCCSRAASATCQS-------------------LCWRAFRADWEA--ASIQLESECLPASAEGELRRCLEDADEPCELGCTGLSYCSN**

**Beetle**  **SRIERVGMDSAKSHCCPRAASHSCQK-------------------LCTKTFTKFWST--SWDEFYNRCLTQVMEENLRNCIDEVDEPCELGCDGLSFCTN**

**Barbel**  **ESPPIAKMDYAKLHCCFKANTSHCRN-------------------MCLEIS-TNWG-TQTWQEFDQHCEYNPAEMDLITCLADVREPCQLGCKDLSYCTN**

**Platyfish**  **ETPPSSQMDCAKLHCCSKANTSVCRE-------------------KCHQIS-THWG-SQTWQDFDQLCEYNPMEMELINCLADVREPCQLGCKDLTYCTN**

**Zebrafish**  **ESPPIAKMDSAKLHCCFKANTSICRN-------------------MCVEIS-TSWG-TQSWQEFDQHCEYNPVEMDLITCLADVREPCQLGCKELSYCTN**

**Xenopus**  **APPPSAGLDGAELHCCSKANSSNCRD-------------------LCTKLYSTSWGNTQAWQDFEFACEYNPLEAPMLTCLADVREPCQLGCRNLTFCTN**

**Chicken**  **HPPPSTGLDGAKLHCCSKANSSTCRE-------------------LCTKLYSTSWGSSQSWQEFDRFCEYNAVEVSMLTCLADVREPCQLGCRNLSYCTN**

**Aligator**  **HPPPSTGLDGAKLHCCSKANTSTCRE-------------------LCTKLYSTSWGNSQSWQEFDRFCEYNPVEVSMLTCLADVREPCQLGCRNLTYCTN**

**Eagle**  **HPPPSTGLDGAKLHCCSKANSSTCRE-------------------LCTKLYSTSWGNSQSWQEFDRFCEYNAVEVSMLTCLADVREPCQLGCRNLTYCTN**

**Spider**  **IQSEKVGMDSAKLQCCFRAATVNCQR-------------------LCIETFSNQWT--SSWSDFDRICQYQLAESEMIRCLTEVDEPCELGCEGMKYCTN**

**Placozoa**  **SCCSRTTLKQCASTCRRALMNAKYSDLEKLYYIRRKCR------KNSILLFNQTLIPPYDFDRYNRKCSVAE-EGKLNTCLLDVVRPCQLGCRNLTFCSN**

**Cod**  **EPLPPAKMDRAKLHCCSKANTSLCRD-------------------MCQKVS-TNWG-SQTWQEFHQLCEYNVVETQLIACLADVREPCQLGCTGLSYCSN**

**Sea Lamprey** **AASSPTGLDGAKLQCCSR---PACQG----------------CEDLCVGLFSMSWGSAQSWQEFERECEYRLQEPSMMGCLADVREPCQLGCRDLNYCTN**

**Tetradon 1**  **ETPHPAKMDCAKLHCCSKANTSLCRD-------------------MCQEVS-TKWG-SQTWQDFDQLCEYNPVETELTECLADVREPCQLGCKGLSYCTN**

510 520 530 540 550 560 570 580 590 600

....|....|....|....|....|....|....|....|....|....|....|....|....|....|....|....|....|....|....|....|

**Mouse**  **FNNR------------PTELFRSCTAQSDQGAMSDMKLWEKG--SIKMPFISIPVLDIKTCQPEMWKAVACSLQIKPCHSKSRGSIICK-----------**

**JPlamprey 2** **FNNRG----------RPTELFRGCNAQADQGALNDIRLWERG--TIQMPFLIIPVKDIRRCQPETWKAIACVLQIKPCLGSSPVAIICRGRPTELFRGCN**

**JPlamprey 1** **FNNRRSLSLTRSCAARPTELFRSCNAQADQGALNDMRLWEHG--AISMPFLSIPVRDVRRCRPEAWKAIACVLQVKPCHGATPSPIICR-----------**

**Tetradon 2**  **--------------------------------MSDFKLWSNG--TIKMPYMNIPVLDIRKCLPDMWKAVACSLQIKPCHIKSRGSLICK-----------**

**Bf98411**  **FNNR------------PTELFRSCASRSDEDAEQYMSDWQGG--IINMPVMSIPVRDVQECEPDMWKAIACTLQIKPCHSQSHGNRICK-----------**

**Stickleback** **FNNR------------PTELFRSCNVQSDQGAMSDMRLWSNG--TIKMPVMNIPVLDIRKCLPDMWKAVACSLQIKPCHSKSRGSVICK-----------**

**Bf215943**  **FNNR------------PTELFRSCASRSDEDAEQYMSDWQGG--IINMPVMSIPVRDVQECEPDMWKAIACTLQIKPCHSQSHGNRICK-----------**

**Aphid**  **FNNR------------PTELFRSCSPQTDDAAHYDVTLWRQRGTITLFN-YELPLKNITKCLPDSWKAVACILQIRPCTRQSHVNKICR-----------**

**Turtle**  **FNNR------------PTELFRSCNAQSDQGAMNDMKLWEKG--SIKMPFINIPVLDIKKCQPETWKAIACSLQIKPCHSKSRGSIICK-----------**

**Fruit fly**  **FNNR------------PTELFRSCTPEHDAAAREDWQLLQQRGTVRVLG-QELFIKNTSRCAPDKWRALVCALQLKPCTRVGLFNGICS-----------**

**Honybee**  **FNDR------------STTLFRSCSATTDEAAKFEAHHWSRGGIIRGLS---LPIRVAATCPPETLRAAACLLQLRPCETKSHETRLCR-----------**

**Ant**  **FNDR------------PTTLFRTCTSTADEAAKWEADHWSRGGIIRGLG---VPVRAAASCPAETLRAAACLLQLRPCETRIHETRLCR-----------**

**Tetra**  **FNNR------------PTELFRSCNVQSDQGAMNDIKLWSNG--TIRMPVMNIPVLDIRKCQPEMWKAVACALQIKPCYSKSRGSVICK-----------**

**Sea urchin**  **FNNR------------PTDLFRSCSIRSDEGAAYDMQLWSEG--LIRMPFMNIPVLNIAECESDMWKTIACTLQVKPCHRKTHTNMICK-----------**

**Mosquito**  **FNNR------------PTELFRSCRAQSDMAARSDVRQWRQQGYIPFPGGVQLPIRNQTECSFEIWRSIACAAQIKPCTKDYHTNHICR-----------**

**Cavefish**  **FNNR------------PTELFRSCNVQSDQGAMNDIKLWSNG--TIRMPVMNIPVLDIRKCQPEMWKAVACALQIKPCYSKSRGSVICK-----------**

**Rat**  **FNNR------------PTELFRSCTAQSDQGAMSDMRLWEKG--SIKMPFISIPVLDIRTCQPDLWKAVACSLQIKPCHRKSRGSIICK-----------**

**Medaka**  **FNNR------------PTELFRSCNIQSDQGAMNDMKLWSNG--TIKMPFMNIPVLDIRKCLPNMWKAVACSLQIKPCHSRSRGSLICK-----------**

**Human**  **FNNR------------PTELFRSCNAQSDQGAMNDMKLWEKG--SIKMPFINIPVLDIKKCQPEMWKAIACSLQIKPCHSKSRGSIICK-----------**

**Tilapia**  **FNNR------------PTELFRSCNIQSDQGAMNDIKLWSNG--TIKMPFMNIPVLDIRKCQPHMWKAVACSLQIKPCHSRSRGSVICK-----------**

**Lizard**  **FNNR------------PTELFRSCNAQSDQGAMNDMKLWEKG--SIKMPFINIPVLDIKKCQPETWKAIACLLQIKPCHSKSRGSIICK-----------**

**Shark**  **FNNR------------PTELFRSCNAQSDQGAKNDMKLWEKG--TIKMPFMNIPVQNIKTCQPEMWKAVACSLQIKPCHSKSGGSVICK-----------**

**Fugu**  **FNNR------------PTELFRSCNVQSDQGALNDIKLWSNG--TIKMPFMNIPVLDIRKCLPDMWKAVACSLQIKPCHK-SRGSVICK-----------**

**Coelacanth**  **FNNR------------PTELFRSCNAQSDQGAMNDMKLWENG--SIKMPFMNIPVLNIKTCQPERWKAVACSLQIKPCHSKSRGSVICK-----------**

**Spotted gar** **FNNR------------PTELFRSCNAQSDQGAMSDIKLWEEG--SIKMPFMNIPVLDIRKCRPEMWKAVACALQIKPCYSKSRGSVICK-----------**

**Fopius**  **FNNR------------PTTLFRTCSAESDITAKQEVDRWARGAVIRGLG---VPVRVDPSCPAESLKAAVCFLQLRPCEPQVHETRLCR-----------**

**Beetle**  **FNNR------------PTELFRSCNTQADEAARNDVSVWQFQNKLTLPG-LILPLKNISQCSPNVWKTVACTLQIKPCSRHSHANQICR-----------**

**Barbel**  **FNNR------------PTELFRSCNVQSDQGAMNDFRLWSNG--TIKMPFMNIPVLDISRCQPEMWKTVACALQIKPCYSKSRGSVICR-----------**

**Platyfish**  **FNNR------------PTELFRSCNMQSDQGAMNDIKLWSNG--TIKMPFMNIPVLDIRKCLPNMWKAVACSLQIKPCHSKSRGSVICK-----------**

**Zebrafish**  **FNNR------------PTELFRSCNVQSDQGALNDFKLWSNG--SIRMPLMNIPVLDIRRCRPEMWKTVACALQIKPCYSRSRGSVICR-----------**

**Xenopus**  **FNNR------------PTELFRSCNIQSDQGAMNDMKLWEKG--SIKMPFMNIPVLDIKKCHPEMWKAIACSLQIKPCNSKSRGSIICK-----------**

**Chicken**  **FNNR------------PTELFRSCNSQSDQGAMNDMKLWEKG--SIKMPFINIPVLDINKCQPEMWKAIACSLQIKPCHSKSRGSIICK-----------**

**Aligator**  **FNNR------------PTELFRSCNAQSDQGAMNDMKLWEKG--SIKMPFINIPVLNIKKCQPEMWKAIACSLQIKPCHSKSRGSIICK-----------**

**Eagle**  **FNNR------------PTELFRSCNTQSDQGAMNDMKLWEKG--SIKMPFINIPVLDIKKCQPEMWKAIACSLQIKPCHSKSRGSIICK-----------**

**Spider**  **FNFR------------PTELFRSCDQRADKAAQHDIEHWENG--IIHLPMMEIPVLKISRCHSEVWKAIACALQIKPCHPIAHINRICK-----------**

**Placozoa**  **FNRP-------------LTYFRNCDSSSDSYAQRVYNNIKST-------LASAASIDVETCYPNIWKAMACILQLRPCHTRFHGAYICK-----------**

**Cod**  **FNNR------------PTELFRSCSVQSDQGAMNDIKLWSNG--TIKMPFMNIPVLHIRKCQPEMWTAVACALQIKPCHSKSRGSVICK-----------**

**Sea Lamprey** **FNNR------------PTELFRGCNAQADQGALNDIRLWERG--TIQMPFLIIPVKDIRRCQPETWKAIACVLQVKPCLGSSPVAIICR-----------**

**Tetradon 1**  **FNNR------------PTELFRSCNVQSDQGAMSDFKLWSNG--TIKMPYMNIPVLDIRKCLPDMWKAVACSLQIKPCHIKSRGSLICK-----------**

610 620 630 640 650 660 670 680 690 700

....|....|....|....|....|....|....|....|....|....|....|....|....|....|....|....|....|....|....|....|

**Mouse**  **------------------------------------------------------SDCVEILKKCGDQNKFPEE-HTAESICE-FLSPADDLESCIPLDTY**

**JPlamprey 2** **AQADQGALNDIRLWERGTIQMPFLIIPVKDIRRCQPETWKAIACVLQIKPCLGSSPVAIICRQCGDMDRFPAG-HSADTICD-ILSPTDEPEQCIPLSRY**

**JPlamprey 1** **-----------------------------------------------------RSDCVDILQQCGDSSRFSEG-QTAEGICE-ALSPSDDADACIPLSRY**

**Tetradon 2**  **------------------------------------------------------SDCVDILTQCGDRRRFHEG-QTPERICE-LLSPVDDPERCIPLHRY**

**Bf98411**  **------------------------------------------------------ADCVNILTRCGDQARFPMG-TTVDSLCN-ALSPQDEDDHCITLAPY**

**Stickleback** **------------------------------------------------------SDCLDILTQCGDRKRFHEG-QTPERICE-LLSPIDDPDRCIPLHKY**

**Bf215943**  **------------------------------------------------------ADCVNILTRCGDQARFPMG-TTVDSLCN-ALSPQDEDDHCITLAPY**

**Aphid**  **------------------------------------------------------DDCLELLSTCLDWSNSAPG-LSANALCA-RLSPNSG--PCVPLGAF**

**Turtle**  **------------------------------------------------------SDCVEILKKCGDQNKFPEG-HTAESICE-LLSPTDDFENCIPLDTY**

**Fruit fly**  **------------------------------------------------------EDCHELLDECLDWTLQHQRPKDICSRLK-EKDDDDSPLPCISLESY**

**Honybee**  **------------------------------------------------------EDCLELMASCVDWSAITGP-HTAATLCA-KLSPAKSDAPCVSLKPF**

**Ant**  **------------------------------------------------------EDCLELMASCVDWGAITGP-HTAATLCA-KLSPGRPDAPCVSLRPY**

**Tetra**  **------------------------------------------------------SDCVDILTHCGDFTRFPEG-QTPERICD-LLSPTDDPERCIPLHRY**

**Sea urchin**  **------------------------------------------------------SDCIHILSKCIDHTRLAKG-VDPVGLCN-ILSPFDDNAPCVSLNPF**

**Mosquito**  **------------------------------------------------------QDCAEVLKNCVDWMRMPEG-HSVSSLCQ-RLSPQNSSMPCISMGTF**

**Cavefish**  **------------------------------------------------------SDCVDILTHCGDFTRFPEG-QTPERICD-LLSPTDDPERCIPLHRY**

**Rat**  **------------------------------------------------------SDCVEILKKCGDQNKFPEA-HTAESICE-FLSPADDVENCIPLDTY**

**Medaka**  **------------------------------------------------------SDCVSILTRCGDSQRFHEG-QTAERICE-LLSPIDDPDRCIPLHKY**

**Human**  **------------------------------------------------------SDCVEILKKCGDQNKFPED-HTAESICE-LLSPTDDLKNCIPLDTY**

**Tilapia**  **------------------------------------------------------SDCVDILTQCGDTKRFHEG-QTPERICE-LLSPIDDPEHCIPLYKY**

**Lizard**  **------------------------------------------------------SDCVEILKKCGDLSKFPEG-HMAESVCE-LLSPTDDLETCIPLDTY**

**Shark**  **------------------------------------------------------SDCLDILRNCGDHSKFPDE-QTAETICD-LLSPSDDSEYCIPLSKY**

**Fugu**  **------------------------------------------------------SDCVDILTQCGDKRRFHEG-QTPERICE-LLSPMDDPERCIPLRRY**

**Coelacanth**  **------------------------------------------------------SDCVDILKSCGDHSKFPEG-QTAETICE-LLSPTDDKEYCIPLESY**

**Spotted gar** **------------------------------------------------------SDCVDILTHCGDHSKFNEG-QSPETICD-LLSPTDDPERCIPLQRY**

**Fopius**  **------------------------------------------------------NDCIDLLTNCVDWSLTPGT-QSAISLCS-HLSPPRPEMPCISLKPF**

**Beetle**  **------------------------------------------------------DVCLDILSKCVDWTRISPE-HSAETICA-SLSPEDPNVSCIRLQNF**

**Barbel**  **------------------------------------------------------SDCVDILTQCGDGKRFLEG-QTPERICD-LLSATDDPERCIPLHRY**

**Platyfish**  **------------------------------------------------------SDCVDILTKCGDKKRFHEG-QTPERLCE-VLSPIDDPERCIPLHRY**

**Zebrafish**  **------------------------------------------------------SDCVEILRQCGDRRRFAEA-QTPERICD-LLSPTDDPERCIPLNRY**

**Xenopus**  **------------------------------------------------------TDCIEILTKCGDHSRFPES-HTAESICE-LLSPSDENEDCIPLDSY**

**Chicken**  **------------------------------------------------------SDCVEILKKCGDHNKFPEG-HTAESICE-LLSPTDDLENCIPLDTY**

**Aligator**  **------------------------------------------------------SDCVEILKKCGDQSKFPEG-HTAESICE-LLSPMDDLESCIPLDTY**

**Eagle**  **------------------------------------------------------SDCVEILKKCGDHNKFPEG-HSAESICE-LLSPTDDLENCIPLDTY**

**Spider**  **------------------------------------------------------ADCIDILNSCVDRSRLLHN-QNPVTLCE-ILSPPGNDTPCISLSPY**

**Placozoa**  **------------------------------------------------------SDCKQIIRSCSHN--VTNN--IVESLCNNLIATNSAYPHCIRLEDY**

**Cod**  **------------------------------------------------------SDCVDILTQCGDLKRFHQG-QTPERICE-LLSXXXXXXXXXXXXXX**

**Sea Lamprey** **------------------------------------------------------SDCVDILEQCGDMDRFPAG-HSADTICD-ILSPTDEPEQCIPLSRY**

**Tetradon 1**  **------------------------------------------------------SDCVDILTQCGDRRRFHEG-QTPERICE-LLSPVDDPERCIPLHRY**

710 720 730 740 750 760 770 780 790 800

....|....|....|....|....|....|....|....|....|....|....|....|....|....|....|....|....|....|....|....|

**Mouse**  **LRPSALGN------------------IIEEVTHPCNPNPC-----PANELCEVNRK-GCPSADPCLPYSCVQ--------GCKLGEASDFIVRQGTLIQV**

**JPlamprey 2** **L---------------------------EEVVHPCNPNPC-----PTSHLCEVNRK-GCTPGQDCLPYLCVPGERLYLCIGCKLGEASDFLVRKDSMVRV**

**JPlamprey 1** **L---------------------------DEVVHPCNPSPC-----PGNQLCEVNRR-GCSPGVDCLPYVCAPG-------GCKLGEASDFLVRRGSVARV**

**Tetradon 2**  **LGPSVLDSD-----------------RAEEVVHPCNPNPC-----PSNHVCQVNRR-GCLDALRCQPYICL-------------GEASDFLVQLDARVQV**

**Bf98411**  **LVPSQHMS------------------RLDEVTNPCNPSPC-----MES-VCHVNRARENCTGSSCPQHICKQ--------GCPMGEASDYVVASGAVVRV**

**Stickleback** **LTPSSLGNS-----------------VVDEVTHPCNPNPC-----PSNHLCQVNRR-GCLDDLHCQPFLCVPA-------GCKLGEASEFLVQQDARIEV**

**Bf215943**  **LVPSQHMS------------------RLDEVTNPCNPSPC-----MES-VCHVNRARENCTGSSCPQHICKQ--------GCPMGEASDYVVAAGAVVRV**

**Aphid**  **AELGAPRPPPPRLQTELQHAAVEDLP---EVTTPCKRNPCA-----PGSVCLVNRG--CRIGRGCKPYRCVA--------GCKAGEVSHFLVPEDSYARI**

**Turtle**  **LSPSSLGN------------------IVEEVTHPCNPNPC-----AANQLCEVNRK-GCQSGEPCLPYFCVQ--------GCKLGEASDFIVRQGTLIQV**

**Fruit fly**  **LK--PGDAS-----PEEFQG----------ITSPCAQKPCN-----GSEVCILQRG--GNQG-----YSCIP--------GCNLGQDSKLFVPFGSYVRL**

**Honybee**  **LEDPQDNES----VVRLEED----------ITTPCKINPCA-----QGEICQLLH-----HGRQA--YRCVP--------ACFLGKMSKQLVPVGSWAQI**

**Ant**  **LDDSQDDET----VIHPEED----------ITTPCKRNPCP-----QGQLCVLQP-----NGAKI--YRCVP--------ACSLGEMSKQLVPVGSWIQI**

**Tetra**  **LTPSPFDN------------------IIEEVIHPCNPNPC-----PSNHLCEVNRK-GCHAGQDCLPYFCVP--------GCKLGEASEFLVHMEARIQV**

**Sea urchin**  **LDPSQHHA------------------SYLDITTPCQSDPCNNDTEVANGVCSVNRK-MCGHREVCQHHTCNR--------GCRLGEASSFLVPIGSYVRL**

**Mosquito**  **QLGVPAGGSGSLVMPSMAAGDTREATGEVALVSPCKGNPCN-----STQVCVANRD--GTYG-----YRCVD--------GCPLGEASSYLVPVGTFVRI**

**Cavefish**  **LTPSPFDN------------------IIEEVIHPCNPNPC-----PSNHLCEVNRK-GCHAGQDCLPYFCVP--------GCKLGEASEFLVHMEARIQV**

**Rat**  **LRPSALGN------------------IIEEVTHPCNPNPC-----PANELCEVNRK-GCPSGDPCLPYSCVQ--------GCKLGEASDFIVRQGALIQV**

**Medaka**  **LTPSSIGET-----------------VVKEVIHTCNPNPC-----PSNYLCQVNRK-GCLDEFDCQPFLCVP--------GCKLGEASEFLVPRNSRVQV**

**Human**  **LRPSTLGN------------------IVEEVTHPCNPNPC-----PANELCEVNRK-GCPSGDPCLPYFCVQ--------GCKLGEASDFIVRQGTLIQV**

**Tilapia**  **LTPSSLGTN-----------------TVEEVIHPCNPNPC-----PSNHICQVNRK-GCLDELNCQPYLCVP--------GCKMGEASEFLVQQDARIQV**

**Lizard**  **LRPSTLDN------------------IVEDVTHPCNPNPC-----AANELCEVNRK-GCQPDEPCLPYFCVQ--------GCKLGEASDFIVRQGTLIQV**

**Shark**  **LAPSPLDS------------------IIEEVIHPCNPNPC-----ANNQVCEVNRK-GCLAGQECLPFLCVQ--------GCKLGEASDFLVRLGNRIQL**

**Fugu**  **LTPSTLGNN-----------------DIEEVIHPCNPNPC-----PSNHVCQVNRK-GCLDGLSCQPYVCVA--------GCKLCEASDFLVQLDARVQV**

**Coelacanth**  **LRPSLIDN------------------IVD-VIHPCNPSPC-----PTNQLCVVNRK-GCQAGEDCLPYFCVP--------GCKLGEASDFLVHQEAHIQV**

**Spotted gar** **LSPSSLGD------------------IIEEVIHPCNPNPC-----PSNQLCEVNRK-GCQPGQECLPYFCVQ--------GCKLGEASEFLVHRRALIQV**

**Fopius**  **IS------P----ILPEKGR----------ITNPCRDSPCG-----KSEVCIPQHN--MSDNSTA--YECLP--------GCVLGEMSHLHVPQNTWVQI**

**Beetle**  **LY--PSETS----VQRISEQ----------VFSPCKGNPCE-----PNEVCLLNRK--CIHGTNCLPYKCVP--------GCKLGEVSEYKVPHGTYVWI**

**Barbel**  **LTPSPFQN------------------TAEEVIHPCNPSPC-----PSSHLCEVNRK-GCHPGHDCLPYFCVP--------GCKLGEASEFLVPLDSRIQV**

**Platyfish**  **LTQSPLSTT-----------------IVEEIIHPCNPNPC-----QSNHLCQVNRK-GCLDEINCQPYLCVP--------GCKMGEASEFLVQQDTLIQV**

**Zebrafish**  **LTASELES------------------SVEEVIHPCNPNPC-----PSSHLCHVNRK-GCHVGHDCLPYYCVP--------GCKLGEASEFLVPADARLQV**

**Xenopus**  **LKSSPLDN------------------AIEEVTHPCNPNPC-----PANHLCEVNRK-GCLPGEPCLPYFCSQ--------GCKLGEASDFLVRHGVLIQM**

**Chicken**  **LSPSSLGN------------------IVEDVTHPCNPNPC-----AANQLCEVNRK-GCQSGELCLPYLCVP--------GCKLGEASDFIVRQGTLIQV**

**Aligator**  **LSPSSLGN------------------IVEDVTHPCNPNPC-----AANQLCEVNRK-GCHSGEPCLPYLCVP--------GCKLGEASDFIVRQGTLIQV**

**Eagle**  **LSPSSLGN------------------IVEDVTHPCNPNPC-----AANQLCEVNRK-GCQSGELCLPYLCVP--------GCKLGEASDFIVRQGTLIQV**

**Spider**  **LVESKHTQ------------------TSTEVSHPCKPNQC-----SPNGVCMVERN--CRIGQPCRSYACVP--------GCRMGDMSQLIVPRSSYVRV**

**Placozoa**  **IGNTTEISTDS-----------WTALNLTERLSPCHPNPCG------SSKCEIINR-CLVDDKDCVSYRCLS--------DCPLGVASHYFIKKGSYIKL**

**Cod**  **XXPSSLAHS-----------------ARGEVTHPCNPNPC-----PSNHLCQVNRK-GCPDDFNCQPYFCVPA-------GCKLGDASDFLVPMDTRLQV**

**Sea Lamprey** **LRVASLGF------------------CSEEVVHPCNPNPC-----PTSHLCEVNRK-GCTPGQDCLPYLCVPG--------CKLGEASDFLVRKDSMVRV**

**Tetradon 1**  **LGPSVLDSD-----------------RAEEVVHPCNPNPC-----PSNHVCQVNRR-GCLDALRCQPYICVPA-------GCKLGEASDFLVQLDARVQV**

810 820 830 840 850 860 870 880 890 900

....|....|....|....|....|....|....|....|....|....|....|....|....|....|....|....|....|....|....|....|

**Mouse**  **PSSAGEVGCYKICS---------CGQSGLLENCMEMHCIDLQKSCIVG--------------------------------------------GKRKS---**

**JPlamprey 2** **PVASGEVGCYRLCS---------CGTSGRLEDCAETPCLDT-RSCIVG--------------------------------------------GQRKSCLR**

**JPlamprey 1** **PVASGEAGCYRLCE---------CGEGGRLENCKEMPCIDSQRSCIVG--------------------------------------------GKREG--N**

**Tetradon 2**  **PTRTGPAGCYEVCS---------CGPSGRLENCVEMPCLDIHKPCVVG--------------------------------------------GQRRS---**

**Bf98411**  **PVASGDPGCYRVCR---------CAEDRRLGDCTTLSCIP-MDTCLVGGQIKGRTVPPCHVSPWTPVWWGDRLKVRDCTTLSCIPMDTCLVGGQIKD---**

**Stickleback** **PTRTGLTGCHEVCT---------CGPSGRLENCAEMPCVDTDEPCIVG--------------------------------------------GERKN---**

**Bf215943**  **PVASGDPGCYRVCQ---------CAVDRRLGDCTTLSCIP-MDTCLVG--------------------------------------------GQIKD---**

**Aphid**  **PTFNGQKGCAK---------ICLCTKRG-IEKCQQVPCSQIQSCWLVGKP--------------------------------------------------**

**Turtle**  **PSSAGDVGCFKICT---------CGQSGLLENCMEMHCVDLQKSCIVG--------------------------------------------GQRKS---**

**Fruit fly**  **GKSNLHKKLEVGQFPLAEHIVCSCGLQGRLEQCQPLPSYMHAHCTLPGARS-------------------------------------------------**

**Honybee**  **P-SFN-QASHQ---------ICQCTPYG-LDKCKMLNYVKFNPCSIHNQI--------------------------------------------------**

**Ant**  **P-RFDQQGCLN---------ICQCTTRG-LEKCRTPNCFNFKSCWVHDRF--------------------------------------------------**

**Tetra**  **PVRSGQAGCYEVCT---------CGQSGRLENCVEMPCIDTTQTCVSG--------------------------------------------EQRKI---**

**Sea urchin**  **PDSGENLDCYQSCV---------CGPTGQLEHCQPLQCERSEMCLISG---------------------------------------------QMKE---**

**Mosquito**  **PVSVTQKGCLK---------VCRCGESGRIEKCQPLPCISYNACSLAGRR--------------------------------------------------**

**Cavefish**  **PVRSGQAGCYEVCT---------CGQSGRLENCVEMPCIDTTQTCVSG--------------------------------------------EQRKI---**

**Rat**  **PSSAGEVGCYKICS---------CGQSGLLENCMEMHCIDLQKSCIVG--------------------------------------------GKRKS---**

**Medaka**  **PT--GLPGCYEVCT---------CGPSGRLENCMEMHCEDSDLPPISE--------------------------------------------AQSKG---**

**Human**  **PSSAGEVGCYKICS---------CGQSGLLENCMEMHCIDLQKSCIVG--------------------------------------------GKRKS---**

**Tilapia**  **PTRTDPTVCFEVCS---------CGPSGRLENCVEMPCMDTSKPCIVG--------------------------------------------GQRKS---**

**Lizard**  **PSSSGEVGCYKICT---------CSQSGLLENCMEMHCVDHQKSCIVG--------------------------------------------GQRKS---**

**Shark**  **PVTSGEMGCYKVCT---------CGPSGRLETCMEMPCIDVQKTCIVG--------------------------------------------GQRKN---**

**Fugu**  **PTRAGPAGCYEVCS---------CGPSGRLENCVETPCVD-IKPCMVG--------------------------------------------GQRRS---**

**Coelacanth**  **PSSTRDN-CYKICT---------CGQSGRLENCKEMPCVEKERTCIVG--------------------------------------------AQRKD---**

**Spotted gar** **PSPKGEVGCYEICT---------CGQSGHLENCVDMPCVDTEKTCIVG--------------------------------------------GQRQS---**

**Fopius**  **P-REDQIGCLR---------ICQCTQNG-VEKCRNLNCFPVNSCFIQNRY--------------------------------------------------**

**Beetle**  **PNDNNNKQQSCLK-------ICKCN-AGKIEECQPLLCVKLKPCLMGTAM--------------------------------------------------**

**Barbel**  **PTRSGQVGCYEVCT---------CGQSGRLQNCAEMPCFDTSKTCFIG--------------------------------------------GQRKS---**

**Platyfish**  **PTRDGAPSCYEMCS---------CGTSGRLENCVEIPCVETDKGCIVE--------------------------------------------GQRKN---**

**Zebrafish**  **PVHSAQPGCYEVCV---------CGQSGRLENCAEMPCFDTSKSCQIA--------------------------------------------GQRRS---**

**Xenopus**  **PS--GAAGCYKICT---------CGQSGTLENCLDTQCVDLQKSCIVG--------------------------------------------GQRKN---**

**Chicken**  **PSSAGDVGCYKICT---------CGHTGLLENCVEMHCVDLQKSCIVG--------------------------------------------GQKKS---**

**Aligator**  **PSSAVDVGCYKICM---------CGHSGLLENCVEMHCVDLQKSCIVG--------------------------------------------GERKS---**

**Eagle**  **PSSAGDVGCYKICT---------CGHSGLLENCMEMRCVDLQKSCIVG--------------------------------------------GQRKS---**

**Spider**  **PSNTHGPRCHMVCY---------CNQDNLLEDCMTQPCLSTEHCWHEG---------------------------------------------RRYN---**

**Placozoa**  **LATPGIERCHMICK--------CFANTSMIDQCYSLPCNSEQSCRIAG----------------------------------------------KKT---**

**Cod**  **PPGGGQADCFEVCV---------CGASGRLEDCKEMPCVDTSKACVVG--------------------------------------------GQRKX---**

**Sea Lamprey** **PVASGEVGCYRLCS---------CGTSGRLEDCAETPCLDT-RSCIVG--------------------------------------------GQRKS---**

**Tetradon 1**  **PTRTGPAGCYEVCS---------CGPSGRLENCVEMPCLDIHKPCVVG--------------------------------------------GQRRS---**

910 920 930 940 950 960 970 980 990 1000

....|....|....|....|....|....|....|....|....|....|....|....|....|....|....|....|....|....|....|....|

**Mouse**  **------HGTSFTIDCNVCSCFAGNLVCSTRLCLSEHSSDDDRRTFTG-----LPCNCADQFVPVCAQNGRTYPSACIARCVG-LQDHQFEFGPCISKNPC**

**JPlamprey 2** **ARVPTGHGTHFRVDCNVCSCFAGELSCSVRQCLSGESSEEDRRRFTGRPTPGLPCNCADQFVPVCAINGRTYPNACIARCTG-LQDSEIEYGTCATRDPC**

**JPlamprey 1** **AAGGSAHGSHFHVDCNVCACYAGRLTCSQRQCFGVASSEEDRRRYTG---AGLPCHCVDQFVPVCAATGRTFPSACVARCAG-LLDSQFEYGTCASRDPC**

**Tetradon 2**  **------PGSTFRVDCRLCSCLAGDSVCSRRRCLGSEGAQP--QHLTG-----LPCDCPDRFVPVCASNGRNYPSACVARCLG-FQDHQFVFGTCGMGNPA**

**Bf98411**  **------HGTSFELDCNICACFAGELTCTKRPCLSLGSTEEERRRYTG-----LPCNCADQFVPVCAHNGRTYPSACLARCAG-LLDHQFEFGTCSSLHPC**

**Stickleback** **------HGASFKIDCHTCSCFAGETTCSTRKCLDLDSSEEDRRRFTG----RLPCNCPDRFVPVCASNGRTYPSACVARCMA-FKDYQFVFGRCRLGNPC**

**Bf215943**  **------HGTSFELDCNICACFAGELTCTKRPCLSLGSTEEERRRYTG-----LPCNCADQFVPVCAHNGRTYPSACLARCAG-LLDHQFEFGTCSSLHPC**

**Aphid**  **----IDHGSTFEMDCNTCNCFAGEITCTKKHCEPATVNAVSRYRHTG-----LPCNCAPHHVPVCGSNGNTYPNSCLAKCAG-LSDVDLKFGTCWNDDPC**

**Turtle**  **------HGTSFNIDCNVCSCFAGNLICSTRQCLNEHNSAEERRMFTG-----LPCNCADQFVPVCGQNGRTYPSACIARCVG-LQDNQFEFGSCISKDPC**

**Fruit fly**  **----YRHGSSFYLECNLCSCFAGEITCTKQQCRLPGF-VDS--GYTS-----LPCNCPAHYVPVCGSNGNTYPSACVAKCH--LPEGDYVYGACNARNAC**

**Honybee**  **----IAHKANFYVECNPCHCFEGEYTCSKKNCG----------EIPS-----LPCDCPPHYVPVCGKLGFTFASGCLATCAR-MSVNDVEFGSCSSRDPC**

**Ant**  **----IQHKTNFYLECNPCHCFEGEFTCSKKSCGEP--------RAPS-----LPCDCPAHYVPVCGRLGFTFASACLAKCAE-LSATEVEFGSCSSRDPC**

**Tetra**  **------HGESFKKDCDSCSCYAGETICSSRKCPSLEHSDDDRHLFRG-----LPCGCPDHFVPVCARNGRTYPSACVARCVG-FQDNQFGYGSCRSSEPC**

**Sea urchin**  **------HASHFHIDCNLCVCFGGNEICSKRQCLTSEMTSEERRRYTG-----LPCDCTDQFVPVCAKNGKTYPSACIAKCVGNFNDEQLEIGTCALNSPC**

**Mosquito**  **----FPHLSHFYVECNVCSCYAEEITCTKRQCRVPGL-TEDR-AFTT-----LPCNCPPHYVPVCGRNGNTYPSACVAKCAG-LSDDDIQFGPCRARNGC**

**Cavefish**  **------HGESFKKDCDSCSCYAGETICSSRKCPSLEHSDDDRHLFRG-----LPCGCPDHFVPVCARNGRTYPSACVARCVG-FQDNQFGYGSCRSSEPC**

**Rat**  **------HGTSFTIDCNVCSCFAGNLVCSTRLCLSEHSSDDDRRAFTG-----LPCNCADQFVPVCAQNGRTYPSACIARCVG-LQDHQFEFGPCISKDPC**

**Medaka**  **----------------------------------------------------LPCDCADHFLPVCASNGRTYPSGCVARCMG-FKDHQFVYGQCHHSDPC**

**Human**  **------HGTSFSIDCNVCSCFAGNLVCSTRLCLSEHSSEDDRRTFTG-----LPCNCADQFVPVCGQNGRTYPSACIARCVG-LQDHQFEFGSCMSKDPC**

**Tilapia**  **------HGTSFKTDCHNCYCFAGETVCSTKECLSSGYTDDSHRHFTG-----LPCSCQDRFVPVCASNGRTYPSACVARCMG-FKDSQFVFGPCHLSKPC**

**Lizard**  **------HGTSFKIDCNVCSCFAGNLICSTRQCLNEYSSDDERRKSTG-----LPCNCEDEFVPMCGHNGRTYPSVCIARCVG-LQDNQLEFGSCIS--PC**

**Shark**  **------HGTSFRVDCNTCSCFAGDLSCSSRQCLSEYSSPEDRNMFTG-----LPCNCADQFVPVCALNGRTYPSACIARCVG-LQDNQFEFGSCASKAPC**

**Fugu**  **------HGSTFRVDCHNCSCYAGTXICPRRECLSPEGSRVDRRHST-------------------------------DRCL------------------C**

**Coelacanth**  **------HGTSFRVDCNVCSCFAGKLICSTRQCVSDGNFVEDQSTFTD-----LPCNCGDKFVPVCAHNGRTYPSACIARCAG-MKDNEFEFGSCALKDPC**

**Spotted gar** **------HGTSFKVDCHTCSCFAGEIVCSTRQCLSKQSSEEDRRRFTG-----LPCGCADQFVPVCAKNGRTYPSACVARCMG-LKDNQFEFGTCRDSDPC**

**Fopius**  **----IPHRTKFYLDCKPCLCFEGEITCSRRPCGDQ--------RLPS-----LPCECPAHYVPVCGRSGVTLASACIAKCRG-LEDDDFEFGSCSFRDPC**

**Beetle**  **------HGTSFNIDCKTCSCFASEDICSKKQCESSAL-TGENTAYTT-----LPCNCVPHYVPVCGKDGNNYPSACLAKCAG-LTDSEIEPAPCEDP--C**

**Barbel**  **------HGVSFRVDCQPCSCFAGESICSSRQCVRADGSDEDRRLFTG-----LPCGCADHFVPVCARNGRTYPSACVARCVG-FKDNQFVFGSCRSIDPC**

**Platyfish**  **------HGTSFRTDCNTCSCFAGETICSTRKCP----TESVHWTSTD-----LPCNCPYRFIPVCASNGRTYPSGCVARCMG-FKDHQYVFGPCHSIKPC**

**Zebrafish**  **------HGSSFRVDCNPCSCFAGDAVCSSRQCVRSDSSEEDRRLFTG-----LPCGCADHFVPVCAGNGRTYPSACVARCVG-FTDSQFVFGSCRSFDPC**

**Xenopus**  **------HGESFKVDCNICSCVAGTLRCSNHQCP---HSEEDRRMFTG-----LPCNCEDQFVPVCGQNGRTYPSACIARCVG-LLDHQFEFGLCSSKDVC**

**Chicken**  **------HGTSFNIDCNVCSCFAGNLICSTRQCLTEHSSEDERQKFTG-----LPCNCVDQFVPVCGQNGRTYPSACIARCVG-LQDNQFEFGSCISKDPC**

**Aligator**  **------HGTSFNIDCNVCSCFAGSLICSTRQCLNEHSSDDERRVFTG-----LPCNCADQFVPVCGQNGRTYPSACIARCVG-LQDYQFEFGTCISKDPC**

**Eagle**  **------HGTSFNIDCNVCSCFAGNLICSTRQCLTEHSSEDERRKFTG-----LPCNCVDQFVPVCGQNGRTYPSACIARCVG-LQDNQFEFGSCISKDPC**

**Spider**  **------HNTQFKAGCKTCFCYDGEVTCSPQICS------------TG-----LPCNCQDHYVPVCGANGKTYPSACLARCVG-LTDDQFEFGACYDSDPC**

**Placozoa**  **----IEHGKQFYDHCNLCGCFNGKITCTKKNCSASVG-----KPIKG-----LPCDCQLKYEPVCGINGQTFGNPCLAQCAG-LGEDEYRTGQCIEIEPC**

**Cod**  **------XGTPFRVDCHPCSCFAGETICSSRQCYSPDSSDQERQRFTG----GLPCGCPDHFVPVCATNGRTYPSACVARCMG-FKDHQFVYGHCRLNEPC**

**Sea Lamprey** **------HGTHFRVDCNICSCFSGELSCSVRQCLSGESSEEDRRRFTG---SSLPCNCADQFVPVCAINGRTYPNACIARCTG-LQDSEIEYGTCATRDPC**

**Tetradon 1**  **------PGSTFRVDCRLCSCLAGDSVCSRRRCLGSEGAQP--QHLTGT---CLPCDCPDRFVPVCASNGRNYPSACVARCLG-FQDHQFVFGTCGMGNTC**

1010 1020 1030 1040 1050 1060 1070 1080 1090 1100

....|....|....|....|....|....|....|....|....|....|....|....|....|....|....|....|....|....|....|....|

**Mouse**  **NPNL---CPKSQ--RCVPKPQVCLTTFDKFGCSQYECVPRQL---TC-DQARDPVCDTDHMEHSNLCTLYQRG--KSLSYRGPCQPFCRA--K--EPVCG**

**JPlamprey 2** **SPSP---CPSGQRR-CVPTRRVCLSSLEQFSCEQFECLSRQP---ACEGQPQEPACDTTNTEHPSLCALYVRG--RSLAYTGPCQ------------VCG**

**JPlamprey 1** **LGNP---CGSRQRCRCIPRRRVCLTSLEPFGCKQFECLHGQL---ACEAASSESACDTADPEHPLLCSLYGRG--KTLPYVGPCQVSAPSS-A--PLVCG**

**Tetradon 2**  **PASP---APGTRG-QCVPRHRVCLSDSSA--CPQYECVGRPG---VCDRNGGELACDTDGVVHLSVCHLQQAG--KRLAYMGLCQEVCRK--P--QQVCG**

**Bf98411**  **QPDP---CPASK--RCIPRPKVCLNGLDQQHCQQYQCVPR-----SCEGEEDDPVCDVDGVQHPNYCQLLAAG--KLLGYHAPCHEKA-CK-V--SPVCG**

**Stickleback** **AGSP---CQRNQ--RCIPKYRVCLSDLSD--CPQYECAGRPG---GCDRSGVEPACDTDGLVHASRCQLLQAG--KTLAYMGPCQEACRK--P--QQVCG**

**Bf215943**  **QPDP---CPASK--RCIPRPKVCLNGLDQQHCQQYQCVPR-----SCEGEEDDPVCDVDGIQHPNYCQLLAAG--KLLGYHAPCHVT--CK-V--SPVCG**

**Aphid**  **ADDN--ACRSDE--RCVPARQVCLSM-LKKSCVQYKCVSEKT---SCEDAPKGVVCDTDGGEHSNLCHLLRSG--KTLAYSGPCL-VGCNSTG---RVCG**

**Turtle**  **NPNP---CTKNQ--RCIPKQQVCLTSFEKFGCSQHECVPRQF---SC-DQLRDPVCDTDNMEYNNLCTLYQKG--KNVSYRGPCQPFCKS--V--EPVCG**

**Fruit fly**  **QAAPPNSCPSGT--QCLDSRKVCLAS-MQRPCLQYVCVNATA--SNCSTFHQGEVCDSQGRTYPNACALLKANPQGQVAYWSACQSSRFNTSP--SPVCG**

**Honybee**  **ASNP---CDTTE--RCVARGRVCLSE-LHKPCRQYECVPLDCD-PRDEA--GGPVCDKENRQHRSVCAMIRAG--ATLGYRGHCL-EGCTLRG---PVCG**

**Ant**  **ASNP---CKSAE--RCIRKPTVCLSR-LHKSCQQYECVPIDCN-PRDES--SGPVCDRENRQYPSVCAMIRAG--ATLSYRGPCL-RGCSLRG---PVCG**

**Tetra**  **SPNP---CQKNQ--RCIPKRRVCLTDMTDYPCLQYECVNRPS---GCDPNQLDPACDTDNLEHANLCLLYQRG--KTLAYMGHCQDACRV--R--QEVCG**

**Sea urchin**  **QSNP---CNLGY--RCVPKRRVCLS-VNFADCPQYDCIITTD---DCDLDTYAPVCDTNHRTHPNMCTLHSLG--GELAYRGRCQMDCHSSIS--QQVCG**

**Mosquito**  **YQVD---CGPLS--VCLPEWNVCLSA-MHKPCPQYRCVNHTS--TNCSSITD--IRDTDLVYHANLCEMVQAG--ATYAYQEKYR-KHCDTSPRRKQVCA**

**Cavefish**  **SPNP---CQKNQ--RCIPKRRVCLTDMTDYPCLQYECVNRPS---GCDPNQLDPACDTDNLEHANLCLLYQRG--KTLAYMGHCQDACRV--R--QEVCG**

**Rat**  **NPNL---CPKSQ--RCVPKPQVCLTTFDKFGCSQYECVPRQL---TC-DQARDPVCDTDHVEHSNLCTLYQRG--KSLSYRGPCQPFCRT--T--EPVCG**

**Medaka**  **RGRS---CPRKH--RCLPKHRVCLSDSSD--CVQFQCVGRPA---SCDKNRLEPVCDTDGLLHPSMCHLQQAG--KTLAYVGHCQEACRK--Q--QKVCG**

**Human**  **NPNP---CQKNQ--RCIPKPQVCLTTFDKFGCSQYECVPRQL---AC-DQVQDPVCDTDHMEHNNLCTLYQRG--KSLSYKGPCQPFCRA--T--EPVCG**

**Tilapia**  **ASKP---CQRNQ--RCIPKYRVCLSDVSD--CPQYECIGHAA---TCDKNSEDPACDTEGMDHRSLCHLHQAG--KTLAYMGRCQEACRK--P--KQVCG**

**Lizard**  **KSNP---CLKNQ--RCIPKPQVCLTSVEKFGCNQYECVPRQF---SCGDQLRDPVCDTDNIEYANLCTLHQKG--KSMSYKGPCQSFCRS--V--DLVCG**

**Shark**  **TPNR---CPKNE--RCIPKPKVCLTGIDLIDCDQYECISRPV---NCELLAIEPVCDTENVEHINLCALYQRS--KTLSYTGPCQDVCR---Y--EAVCG**

**Fugu**  **AAN----------------------------CP---------------------------------------------------QEVCRK--P--RQVCG**

**Coelacanth**  **NPNP---CPRNK--KCIPKHRVCLTNIHMFGCDQFECVSKSV---TCDQEPLETVCDTENVEHPSRCVLYQRG--KMLSYVGSCQPICRS--N--RRVCG**

**Spotted gar** **FPNP---CRKGQ--RCIPKRKVCLTSIDDYPCTQYECLTFSG---PCDHSLVDPVCDIDNMEHPNLCELYKSG--KTLAYIGHCQDACKK--P--NPVCG**

**Fopius**  **AGNP---CGSGE--KCVRRARVCLAP-IYKPCKQYECVSSNCE-VRRKGSVERPVCDKDHREHKSVCDLVKSG--GAIGYWGPCL-RDCSLRG---PVCG**

**Beetle**  **RSHT---CPIGH--KCVPRPQICLFSREHRDCKQYECINGT---TNCHNLPKSPVCSTNNTEFDNSCLLAHHN--AKLAYHGPCL-RNCRHEG---VVCG**

**Barbel**  **FPNP---CQRSQ--RCVPRRQVCLTELSEYPCPQYECVSRPA---GCDQNQLDPVCDTDNMEHANLCLLFQRS--KSLAYMGHCQDACRK--P--REVCG**

**Platyfish**  **SDIT---CQRNQ--RCIPRRRVCLSDSLD--CPQYECVSRPA---VCDRNSMEPACDIEGRVHRSLCHLQQSG--KMLAYMGHCQEACKK--K--KEVCG**

**Zebrafish**  **SPNP---CQRNQ--RCVPRRQVCLTDLSEFPCPQYECVSRPS---SCDQKLLDPVCDTDNMEHANLCVLNLRG--KTLAYSGHCQDACRR--P--REVCA**

**Xenopus**  **NPNP---CSRNE--RCIPKRQVCITSYEKFGCAQYECIPRQL---KC-EHSRDPVCDTENMEHMNLCTLYQRG--RLLSYKGSCQPFCKS--A--EPICG**

**Chicken**  **NPNP---CSKNQ--RCIPKKQVCLTSFGKFECSQHECVPRQL---NC-DQTQDPVCDTDSVEYSNVCTLYQKG--KNLAYRGPCQPFCKS--V--EPVCG**

**Aligator**  **NPNP---CTKNQ--RCIPKKQVCLTSFEKFGCSQYECVPRQF---NC-DQLRDPVCDTDNVEYNNLCTLYQKG--KNISYKGPCQPFCKS--T--EPVCG**

**Eagle**  **NPNP---CNKNQ--RCIPKKQVCLTSFGKFECSQHECVPRQF---NC-DQTRDPVCDTDNVEYSNLCTLYQKG--KSLAYRGPCQPFCKS--V--EPVCG**

**Spider**  **SPNS---CHPYH--RCVPKKRVCLS-IRHRSCKQYDCVNMQH---NCNQLPKFPVCDTDNVEHPNICWMLQRR--KSLAYYGKCMSHCRG--T--GLVCG**

**Placozoa**  **QVFS---CSSGI---CLTDRKICLG-KQFPGCRQYKCEFIKEQNKDPSKRDINFVCDSNNIQHNSTISLIESR--QTFSYSGYCLDKCSTAGK----VCG**

**Cod**  **SSKP---CQRNQ--RCVPKRRVCLSDATDFPCRQYECVVRPA---GCDSS--DPVCDTDGVVHTNLCKLHQSG--KALAYMGQCQEDCRK--P--QPVCG**

**Sea Lamprey** **SPSP---CPSGQR--CVPTRRVCLSSLEQFSCEQFECLSRQP---ACEGQPQEPACDTTNTEHPSLCALYVRG--RSLAYTGPCQPFT-KS-L--IPVSG**

**Tetradon 1**  **SSKP---CPRNQ--RCVPRHRVCLSDSSA--CPQYECVGRPG---VCDRNGGELACDTDGVVHLSVCHLQQAG--KRLAYMGLCQEVCRK--P--QQVCG**

1110 1120 1130 1140 1150 1160 1170 1180 1190 1200

....|....|....|....|....|....|....|....|....|....|....|....|....|....|....|....|....|....|....|....|

**Mouse**  **HNGETYSSVCAAYSDRVAVDYYGPCQAVGVLSEY-SAVAECAAVKCPSLSA-IGCKPIIPPG--------ACCPLCAGMLRVLFDK----EKLDTIAKVT**

**JPlamprey 2** **HDGESYASVCTAYSERVAVDYAGPCQAVGLLSHH-VASPECDAVTCPALPV-ASCKPITPPGCRAMFPTGACCPICAGMMRILWSK----EQVDILARVS**

**JPlamprey 1** **HDGETYASVCAAHSARVAVDYVGPCRAVGLLPRHGAATPECDPVTCPPLPV-AGCQPVTPPGGPGPVPPGACCPLCAGMLRILWSM----EQLDMLARVQ**

**Tetradon 2**  **HNMETYNTVCHALADRVAVGLRGPLSRCGGPLGR-GPGLCMQHCLLSTAVL-PWLPACDPP-------------RGLFMLQILWNK----EAMNTFSKLN**

**Bf98411**  **HNGETYESNCAAHAARVSIDYEGPCLAVGLRPGQ-GKYPECSTVSCP--DS-SLCEGIIPPG--------ACCPVCGGVLAMLYSG----WYVDLVSRQA**

**Stickleback** **HNGETYNSVCDAMADRVALDYEGPCHAVGAVSDA-APDSACSGIPCPPLSA-PGCQPVTPPG--------ACCPICASMLQILWSK----EQMNTFSKLN**

**Bf215943**  **HNGETYESNCAAHAARVSIDYEGPCLAVGLRPGQ-GKYPECSTVSCP--DS-SLCEGIIPPG--------ACCPVCGGVLAMLYSG----WYVDLVSRQA**

**Aphid**  **VDGNTYPSECAAFAESTSVDYSGPCASSGFIGHNGRPYCAAKGAVDCPKLPQQGCQGITPPG--------ACCPKCA-GALQILFSQKQVDRVMHTLKRP**

**Turtle**  **HNGETYNNVCAAYSDRVAVDYYGHCQAVGVVSDY-SLQTECTSVKCPHLSA-TGCKPVVAPG--------ACCPLCAGMLRVLYDK----DKLDTFAKVT**

**Fruit fly**  **INGVTYKSSYAARAEYVLVDYVGRCREVGLLVSDMGRRC---RTVKCPAPVSKHCRLIVPPG--------ACCPLCAGGAFRIIYSRKQFDRAMYGLRAQ**

**Honybee**  **ANGEVYANECAAWAERTVVDYFGPCAAVGLIGDQAKPRCG--DLVQCPRLIEPYCVGVTPPG--------ACCPVCG-GAAKLFYSKKQLDRIYYVMKED**

**Ant**  **INGEVYANECAAWAEKIIVDYQGPCVAVGLIGEEAKPRCG--DVVQCPALVEPYCIGVTPPG--------ACCPVCG-GAARLFYSKKQLERIYYIMDEE**

**Tetra**  **HNGETYSTVCEAFSDRVAVDYHGVCHAMGAVSEF-MTDSGCNAVPCPPLST-QGCNPVTPPG--------ACCPICAGMLQILWNK----KQMNTFAKLN**

**Sea urchin**  **HNGETYSSECEAWSDRTTVDYYGPCQAVGTLTGEEDIEIQCTTVDCPEVTAPDICVAVTPPG--------SCCPICGMY---------------------**

**Mosquito**  **VNGITYRSECEAWSDYSTVDYNGPCQEVGLINPQLEARC---SSVKCPKRKSLGCNPVLPPG--------ACCPVCG-TAIRIVYSRKQIDRGLYALKGT**

**Cavefish**  **HNGETYSTVCEAFSDRVAVDYHGVCHAMGAVSEF-MTDSGCNAVPCPPLST-QGCNPVTPPG--------ACCPICAGMLQILWNK----KQMNTFAKLN**

**Rat**  **HNGETYSSVCAAYSDRVAVDYYGRCQAVGVLSEH-SAVTECAAVKCPSLSA-IGCKPIIPPG--------ACCPLCAGMLRVLFDK----EKLDTIAKVT**

**Medaka**  **HNGETYDTLCHAFSDRVAMDYEGACRAVGLVSGV-THESACSLVSCPPVST-PGCQSITPPG--------ACCPICASMLQILWNK----EKMNSFSELN**

**Human**  **HNGETYSSVCAAYSDRVAVDYYGDCQAVGVLSEH-SSVAECASVKCPSLLA-AGCKPIIPPG--------ACCPLCAGMLRVLFDK----EKLDTIAKVT**

**Tilapia**  **HNGETYNTVCGAYSDRVAVDYEAPCRAVGAVSDV-APDSACSLVSCPSLST-PGCHPVTPPG--------ACCPICASILQILWSK----ERMNTFSKLN**

**Lizard**  **HNGETYSNVCAAYSDRVAVDYSGPCQAVGVLSDY-SYQGECASVTCPRLSD-TGYRPVTPPG--------ACCPLYAGMLRVLYDK----EKLDIFARVT**

**Shark**  **HNGETYNNVCAAYSDRVAVDYYGSCQAVGILSDF-SSHPECSSVTCPSLPS-SRCKPVTPPG--------ACCPLCAGMLRVLWSN----DQLDSLAKLN**

**Fugu**  **HNGETYNTVCDAFSNRVTVDYEGPCHAVGALTDG-APDSACSIVSC-LLPS-PGCRAVTPPG--------ACCPICASMLQILWNK----DGMNTFSKVR**

**Coelacanth**  **HNGETYSNLCAAYSDRVAVDYYGTCQAVGVLSGY-SSHSQCSSVMCPTLSV-SGCKPITPPG--------ACCPLCAGMLRVLFST----DQLNIFSEII**

**Spotted gar** **HNGETYCTVCAAYSDRVAVDYPGRCHAVGVVSEV-STDSGCATINCPALPT-KGCNPVIPPG--------ACCPLCAGMLQILWTK----EQMNVFAKLN**

**Fopius**  **INGEVYANECAAWAENTIMDYFGPCMAVGRAGEG----CG--EAVKCPDIPEG-CPGVIPPG--------ACCRVCG-AAARLFYSRKQVDRIYYMMDEE**

**Beetle**  **FNGRTYISECAAWADMVSVDYVGRCRRVGLIGTTKTKRC---PDVKCQALPDPNCLGVTPPG--------ACCPICG-GTLNLLYSRQQINRALYALDTN**

**Barbel**  **HNGETYSTVCEAFSDRVAVDYQGRCHAVGVVSEF-TPDSGCNAVPCPPLSS-RACNPVTPPG--------ACCPVCAGLLQILWNK----VQMNTFSKLN**

**Platyfish**  **HNGETYNTVCEAFSDRVAVDYEGPCRAVGALSDL-ALDSVCSEVSCSPISS-PGCHPLIPPG--------ACCPICASMLQILWSQ----KQISTFSKLN**

**Zebrafish**  **HNGESYSTVCEAFSERVAVDYQGRCHAVGLESEF-GSDSGCNAVPCPPLAS-DACQPVTPPG--------ACCPVCAGMLRILWNK----AQMNIFAKLN**

**Xenopus**  **HNGETYPNVCSAYSNRVAVDYYGHCQDVGIFSDQ-GLHSECLSIQCPTIPA-TVCKPVTPPG--------ACCPLCAGVLRILFDK----EKLDTFAMAT**

**Chicken**  **HNGETYSSVCAAYSDRVAVDYYGHCQAVGVLSDY-GFHTECAFVKCPQLSA-TGCKPVIAPG--------ACCPLCAGMLRILYDK----DKLDNFARVT**

**Aligator**  **HNGETYGSVCAAYSDRVAIDYYGQCQAVGVLLDY-GFYTECASVKCPSLSS-TGCKPVIAPG--------ACCPLCAGMLRVLYDK----DRLDTFAQVT**

**Eagle**  **HNGETYSSVCAAYSDRVAVDYYGHCQAVGVLSDY-GFHSECAFVKCPQLST-TGCKPVIAPG--------ACCPLCAGMLRILYDK----DKLDTFARVT**

**Spider**  **HNGETYASECAAWAERVSVDYKGACAAVGSKGSD--LRPTSNSSSHWFVIKDLKCQEGSFCQ-------IFLFTFAGAALRLLYSQ----KLSDASVEAI**

**Placozoa**  **TDGNTYIHECAALAAKVLIDYHSECQNVGIYQAP---------------------------------------------YLQLVIS----DELADNNSLA**

**Cod**  **QNSETYNSVCEAFSDRVAVDYEGPCHAVGAGADY-AVGSGCDLVACPPPSH-PHCVPVTPPG--------ACCPVCASVLRVLWNR----EQMNVFAKLD**

**Sea Lamprey** **HPLEKVSGHCILYKTRICVDNP----IRPMTCLF-TISGISKAVVC------ADCAQLHSDTCRTMFPTGACCPICAGMMRILWSK----EQVDILARTA**

**Tetradon 1**  **HNMETYNTVCHALADRVAVDYEGPCHAVGALSDG-APDSACSIVSCPPLSS-PGCRPVTPPG--------ACCPICASMLQILWNK----EAMNTFSKLN**

1210 1220 1230 1240 1250 1260 1270 1280 1290 1300

....|....|....|....|....|....|....|....|....|....|....|....|....|....|....|....|....|....|....|....|

**Mouse**  **S-KKPITVVEILQKVRMHVSVPQCDVFGYLSIESEIVILIIP--VDHYPKALQI--------EACNKEAEKIESLINSDSPTLASHVPLSALIISQVQVS**

**JPlamprey 2** **NNRGPITVSDIVHGLRLHVSVPQCDVFGYLSIEADIIILVLP--VDLKPTALQVRYILSLQIEACSKEAEKIGVLINSGSPVLVSHVPLSALTNAQVYVS**

**JPlamprey 1** **I-------------------------------------------------------------EACSKEAEKIGALVNSGSPVLVSHTPLSALLAAQVLVS**

**Tetradon 2**  **K-NRPETVQDVLQVLRLHVSVPQCDVFGYLSIDHMLVVLVAP--VERQPTPLQM--------EACSKEAEKIDSLINHASPTLVSHVPLSAFLTSEIRTT**

**Bf98411**  **S-IGPITVTQVIQQLRNHVSVPQCDVFGYLSIEGDIIAIVMP--VTPNTTPLQV--------EACNQEAQKVAALINAGSPVIVSHVPLSFLTSASLHLA**

**Stickleback** **K-NQPVTVHDVLQILRLHVSVPQCDVFGYLSIDHELVVLIAP--VEQQPTPLQI--------EACSKEAEKIDALINYASPTLVSHVPLSAFLTSEIQTS**

**Bf215943**  **S-IGPITVTQVIQQLRNHVSVPQCDVFGYLSIEGDIIAIVMP--VTPNTTPLQV--------EACNQEAQKVAALINAGSPVIVSHVPLSFLTSASLHLA**

**Aphid**  **S-VNALNTKSVLRALDRHVLVAECVVRGHLTMYQDLLVLVES--TVRNPTRLQL--------EACIRESEKLSTLVETSSPRVMIDLSLSTLISASPVHE**

**Turtle**  **N-KKPITVLEVLQKIRLHVSVPQCDVFGYLSIESEIVILIIP--VDQNPKPLQI--------EACNKEAEKIESLINSDSPTLASHVPLSALITSQVQVS**

**Fruit fly**  **S-STLLTLQGVLQQLDGLVQVSECQLTGFLTMEVGIFVAIVPSSSIKRPTHLQL--------EACAREAEKISSLINAQSPRITTNLALSCLTVSHLLEP**

**Honybee**  **VDKDTVTLEALLSALARQIQVAQCVLRGMMTPDRDIFIVVQP--TSRRPSTLQL--------RACVTETEKLVTRILERSPKITVEVPLSALIRAEIAHS**

**Ant**  **ADKDSVTLEVLLGALGRQLQVAQCAIRGMMTPDCDIFIVVQP--VAKKPSALQL--------RACVTETEKLVTRITERSPRIAAEVPLGSLTRAEIAHS**

**Tetra**  **R-NQPITVHNILRILRLHISVPQCDIFGYLSIDSEIVILIVP--VDQQPTPLQI--------EACSKEAEKIDSLINSGSPTLVSHVPLSAFLMSELKLS**

**Sea urchin**  **----------------------------------------------------------------------------------------------------**

**Mosquito**  **S-TEYLTLRSILRSLEALLQTVECRLAGQLTFENDIYVIVET--LVKNPSRLQT--------QICAREAERLVTLVRTQSHRITSELNLSALTVATLATA**

**Cavefish**  **R-NQPITVHNILRILRLHISVPQCDIFGYLSIDSEIVILIVP--VDQQPTPLQI--------EACSKEAEKIDSLINSGSPTLVSHVPLSAFLMSELKLS**

**Rat**  **N-KKPITVLEILQKIRMHVSVPQCDVFGYLSIESEIVILIIP--VDHYPKALQI--------EACNKEAEKIESLINSDSPTLASHVPLSALIISQVQVS**

**Medaka**  **K-NQPVTVHDVLQILRLHVSVPQCDVFGYLSLDHQLVVLIVP--VDQHPTPLQV--------EACSKEAEKIQSLINYASPTLVSHVPLSAFLNADIRTS**

**Human**  **N-KKPITVLEILQKIRMHVSVPQCDVFGYFSIESEIVILIIP--VDHYPKALQI--------EACNKEAEKIESLINSDSPTLASHVPLSALIISQVQVS**

**Tilapia**  **K-KQPVTVHDVLQILRPHISVPQCDVFGYLSIDHLLVVIIAP--VDQQPTPLQI--------EACSKEAEKIDSLINYASPTLVSHVPLSAFLTTEIKTS**

**Lizard**  **--KRPITVLEILQKIRLHVSVPQCDVFGYFSIESEIVILIVP--VDQNPKPLQI--------EACNKEAEKIESLINSDSPALASHVPLSALIAAQVEIS**

**Shark**  **D-ARPVSVHDILHTLRLHISVPQCDIFGYLSIESDLVILIIP--VGQDPTTLQI--------EACNKEAEKIDSLINTGSPALMAHVPLSALTTSQVQVS**

**Fugu**  **----------LLSLFYR-----------------------------------------------------------------------------------**

**Coelacanth**  **D-GRPLTVHDILRSIRLHISVPQCDVFGYFSIESEIVILIVP--VDDHPTSLQI--------EACNKEAEKINSLINFNSPTLVSHVPLSALTTSQVQVS**

**Spotted gar** **N-NKPVTIHDILQILRLHISVPQCDIFGYLSIDSEIVILIVP--VDERPTPLQI--------EACNKEAEKIDSLINYGSPTLVSHVPLSAFTVSEVKVS**

**Fopius**  **RDKDAVTLESLLTALGRQIQISQCVLRGGMTPEGDVFIIIQP--TSKSPSLLEH--------RACLAELEKIVTRIAEKSPKAMTEVPLGSLTGGEMIDE**

**Beetle**  **--TDSLTLKAMLKALDRQIQVAQCVLRGYLTVEMDIFVTVQT--TEKYPSKLQL--------EACVQEAEKIASLVNMQSPRIVSEVTLSSLTLANVIHV**

**Barbel**  **R-NQPVTVHDILKILRLHISVPQCDIFGYLSIDSELIVLIVP--VDQQPTPLQI--------EACSKEAEKIDSLLNSASPTLVSQVPLSAFLRSELQLS**

**Platyfish**  **R-NQPVAVYDVLQILRLHISVPQCDVFGYLSIDNRLVVLVAP--VDHQPTLLQV--------EACSKEAEKIDSLINYASPTLVSHVPLSAFLSSEIRTS**

**Zebrafish**  **R-DQPVSLHDILKILRLHVSVPQCDIFGYLSINSEIIILIAP--VDQQPTPLQI--------EACSKEAEKIDSLINSGSPTLVSHVPLSAFLSSELQLS**

**Xenopus**  **K-NTPITVMDILQKIRQHISVPQCDVFGYLSMESDIIILIVP--VDSPPKSIQI--------DACNKEAEKINSLINSDSPTLVSQVPLSALITSEVQIS**

**Chicken**  **N-KKPITVLDILEKLRLHVSVPQCDVFGYLSIESEIVILIIP--VDQKPKPLQI--------EACNKEAEKIESLINSDSPTLASHVPLSALIASQVQVS**

**Aligator**  **N-KKPITVLEILQKIRLHVSVPQCDVFGYLSIESEIVILIIP--VDQNPKPLQI--------EACNKEAEKIESLINSDSPTLASHVPLSALIASQVQVS**

**Eagle**  **N-KKPITVLDILEKIRLHVSVPQCDVFGYLSIESEIVILIIP--VDQNPKPLQI--------EACNKEAEKIESLINSDSPTLASHVPLSALIASQVQVS**

**Spider**  **RDVDPVVIQTIAEKIREHVKVTECEVFAYLSLESDIIVLVMA--ITDSPTKLQV--------EACVREAEKLQALIQQHSPTLLTELPLSFFTTAMMLDP**

**Placozoa**  **ADQTPITVIHLVKEIQNLLTTVYCIVYGHKSFEGDVQIIIKMT---DPPSMTKFR--------ICNKETESLQTMFYNKNLPISGSISLSTVIAVEQHQA**

**Cod**  **R-DRPVTVQDVLQILRLHVSVPQCDLFGYLSIDHELVVVIAH--LDDRPTRLQV--------EACSKEAEKMDSLINYGSPTLVSHAPLSSFLSAETRVP**

**Sea Lamprey** **N-RGPITVSDIVHGLRLHVSVPQCDVFGYLSIEADIIILVLP--VDLKPTALQI--------EACSKEAEKIGVLINSGSPVLVSHVPLSALTNAQVYVS**

**Tetradon 1**  **K-NRPETVQDVLQVLRLHVSVPQCDVFGYLSIDHMLVVLVAP--VERQPTPLQM--------EACSKEAEKIDSLINHASPTLVSHVPLSAFLTSEIRTT**

1310 1320 1330 1340

....|....|....|....|....|....|....|....|....|..

**Mouse**  **SSLPSSAVVGR-PLFHSLLLLLSLGLTVHLLWTRP------------**

**JPlamprey 2** **TGGVSGVSPPPTGPS-FVLLLG-VLMVTCHRVLCS------------**

**JPlamprey 1** **TGCPSAAPPRTPPPGRPTLLLAALLLAAALRLVAPP-----------**

**Tetradon 2**  **SVHSSGGCPPSPLRPGLCFLLG-LLSAAPLLQQL-------------**

**Bf98411**  **AVTMVSTATAAVPSLPALLLCTATALHRLFLHMS-------------**

**Stickleback** **YVRSSGGAPPSPLHPGLCLLLGLVIAAAPVLHQV-------------**

**Bf215943**  **AVTMVSTATAAVPSLPALLLCLATALHRLFLHMS-------------**

**Aphid**  **MVTDGDASDAAPAFDLRAHVAATITSLILTLLIR-------------**

**Turtle**  **CSISSSSIKAV-PVLQTLLLSLILTLQASINNT--------------**

**Fruit fly**  **TPNGATSYGPHAIWILPLLL--LVSRVIIA-----------------**

**Honybee**  **YISSAITIGGYVTMQIFLAIFLYVIF---------------------**

**Ant**  **YVSSAMKIRAWHVTIILFIFMLKITS---------------------**

**Tetra**  **TVSSAAPHSASSLSLLLLCLLLSTLILPFTGRTP-------------**

**Sea urchin**  **-----------------------------------------------**

**Mosquito**  **GNAGRTLYSSLVVVVVAAALGYLVGGASRH-----------------**

**Cavefish**  **TVSSAAPHSASSLSLLLLCLLLSTLILPFTGRTP-------------**

**Rat**  **SSLPSSAVVGRLPPVHSLPLFLSLGLTVHLLQTCP------------**

**Medaka**  **SITSRG-ALRSPPASGLRLLLVLLLTAALALQQV-------------**

**Human**  **SSVPSAGVRAR-PSCHSLLLPLSLGLALHLLWTYN------------**

**Tilapia**  **SIRSSGSTPSSPLTPALCFLLGLLVTPAP------------------**

**Lizard**  **FGISSSCSQVV-PALHLLFWSFVVTFSATQCKT--------------**

**Shark**  **LRGSSGSAMTI-LSTTALLLNLLVSFSGFLGFR--------------**

**Fugu**  **-----------------------------------------------**

**Coelacanth**  **MKDSSCSAKVI-PFSLAIFVSLIITLGNSI-----------------**

**Spotted gar** **TLTSAAPALAMPVYFSLLLSFSFTLFVSL------------------**

**Fopius**  **KPSSASTWRTLTLLLLLPFLIVGIIT---------------------**

**Beetle**  **N-SASSLGATMVFVVLLLRLF--------------------------**

**Barbel**  **TISSAALPPLS----LSLCVFSSSLLLSLTADL--------------**

**Platyfish**  **SIHSGGSLLAPETCYNFILMFSQSLLIYLLLVYC-------------**

**Zebrafish**  **SVRSSSCVSIS----VCVLLLLCSLILTLTSDL--------------**

**Xenopus**  **TTLTSDCNRIC-LSINYIYLYLAISMLYITFNA--------------**

**Chicken**  **FSISSPSVKVG-PVLHCLFISFSFTLLKLMDYI--------------**

**Aligator**  **LSISSPCIKAM-PVLYTLFLSFVFTLLAVIYNT--------------**

**Eagle**  **FSISSPSVKVV-PVLHSLFISLLFTLSALIYYI--------------**

**Spider**  **VTDSAIQLTFNQTYLLVFLLITVITYHIQRT----------------**

**Placozoa**  **TFQSESPVINDNSSSSAYGITMDKKAILFTVLISLGMNALYCNFQML**

**Cod**  **VLHPSGATLLPALLLSVLLLLGLPVQHH-------------------**

**Sea Lamprey** **TGGVSGVSPRPTGPS-FVLLLAKISCMSCGLSIHMHFLSP-------**

**Tetradon 1**  **SVHSSGGCPPSPLRPGLCFLLG-LLSAAP------------------**
